# ACSS1 co-opts acetyl-CoA metabolism to drive DNA repair and undermine radiotherapy efficacy in breast cancer

**DOI:** 10.1038/s41419-025-08300-w

**Published:** 2025-12-18

**Authors:** Xiao Fu, Yingyu Zhu, Xin Lu, Xingjie Gao, Lingbiao Xin, Yuanyuan Ren, Xin Liu, Lin Ge, Jihui Hao, Zhi Yao, Lei Shi, Jie Yang

**Affiliations:** 1 State Key Laboratory of Experimental Hematology, Key Laboratory of Cellular and Molecular Immunology in Tianjin, and Key Laboratory of Immune Microenvironment and Disease (Ministry of Education), The Province and Ministry Co-sponsored Collaborative Innovation Center for Medical Epigenetics, School of Basic Medical Science, Key Laboratory of Breast Cancer Prevention and Therapy (Ministry of Education), Tianjin Medical University, Tianjin, China; 2 Department of Biochemistry and Molecular Biology, Department of Immunology, School of Basic Medical Science, Tianjin Medical University, Tianjin, China; 3 TianJin Medical University General Hospital Medical Laboratory, Tianjin, China; 4 Tianjin Medical University Cancer Institute and Hospital, Tianjin, China

**Keywords:** Breast cancer, Homologous recombination

## Abstract

Breast cancer remains a leading cause of cancer-related mortality worldwide, with radiotherapy serving as a cornerstone of treatment. However, the development of radioresistance significantly compromises therapeutic efficacy and patient outcomes. Through integrative analysis of TCGA and GEO datasets combined with quantitative proteomics, we identified acetyl-CoA synthetase 1 (ACSS1) as a key driver of radioresistance in breast cancer. ACSS1 is aberrantly overexpressed in breast cancer and correlates with poor prognosis following radiotherapy. Functional studies revealed that overexpressed ACSS1 is able to enhance radioresistance both in vitro and in vivo. Mechanistically, ACSS1 amplifies the ionizing radiation (IR)-induced metabolic coupling of pyruvate with ROS for acetate synthesis, which fuels energy production and expands the nuclear acetyl-CoA pool, enabling histone acetylation at DNA damage sites. Such acetylation promotes chromatin relaxation at damage sites, facilitating the recruitment of homologous recombination (HR) repair machinery and ultimately leading to radioresistance. Our findings reveal that ACSS1 orchestrates acetyl-CoA-driven histone acetylation to enhance DNA repair efficiency, highlighting a metabolic-epigenetic crosstalk that sustains radioresistance in breast cancer. Targeting ACSS1 represents a promising therapeutic strategy to sensitize tumours to radiotherapy and improve clinical outcomes in breast cancer patients.

## Introduction

Radiotherapy is a pillar of cancer treatment, achieving durable disease control in solid tumours through the induction of lethal DNA double-strand breaks (DSBs) [1, 2]. Despite technological advances such as stereotactic ablative radiotherapy (SABR) and proton therapy that have improved precision [3–5], therapeutic resistance remains a substantial clinical obstacle, leading to disease recurrence and mortality. Resistance mechanisms are multifaceted, involving tumour microenvironment remodeling [6], antioxidant defense activation [7], and metabolic adaptations [8]. A central mechanism is enhanced DNA repair, particularly via HR and non-homologous end joining (NHEJ). HR, a high-fidelity pathway active in the S/G2 phase, is orchestrated by BRCA1 to mediate DSB resection, whereas the error-prone NHEJ, facilitated by 53BP1, operates throughout the cell cycle [9, 10]. Consequently, targeting key DNA repair proteins—such as ATM/ATR kinases or RAD51—has emerged as a promising radiosensitization strategy. Preclinical and early clinical studies demonstrate that combining ATR inhibitors Berzosertib with radiotherapy significantly enhances tumour response in solid tumours [11]. While these approaches validate the disruption of repair pathways, their broader clinical translation necessitates deeper mechanistic insights into the drivers of repair proficiency.

In breast cancer, radiotherapy achieves sustained locoregional control in a substantial subset of patients, yet therapeutic failure persists with resistance mechanisms demonstrating molecular subtype-specific patterns [12, 13]. Retrospective studies indicate that human epidermal growth factor receptor 2 (HER2)-enriched tumours exhibit pronounced radioresistance, showing higher locoregional recurrence rates compared to other subtypes. In contrast, triple-negative breast cancer derives the greatest survival benefits from radiotherapy [14]. These observations, however, can be confounded by cohort heterogeneity. Combinatorial strategies aim to overcome resistance, with poly(ADP-ribose) polymerase inhibitors including olaparib reducing recurrence risk by 42% in BRCA1/2-mutated breast cancer [15]. However, tumours retaining HR proficiency show limited therapeutic gains, highlighting HR competency as a critical resistance determinant. HR efficiency relies on chromatin relaxation at DSBs, enabled by histone acetylation—a process critically dependent on nuclear acetyl-CoA availability [16]. This poses a significant metabolic challenge following IR: How do resistant tumours sustain sufficient nuclear acetyl-CoA flux to fuel DNA repair?

Emerging evidence reveals that metabolic adaptations in acetate utilization critically enable therapeutic resistance [17]. Acetate, an alternative carbon source, is converted to acetyl-CoA by acetyl-CoA synthetases, with the mitochondrial isoform ACSS1 demonstrating unique adaptive capabilities under nutrient deprivation [18]. ACSS1 dysregulation drives cancer progression by enabling tumour adaptation to hostile microenvironments characterized by hypoxia and nutrient deprivation [19, 20]. Studies demonstrate ACSS1 overexpression in multiple malignancies, where it reprograms metabolism to utilize acetate as an alternative carbon source, maintaining acetyl-CoA pools for critical cellular functions. This metabolic flexibility correlates with enhanced tumour aggressiveness and poor clinical outcomes [21]. Despite its established role in supporting cancer survival, whether and how ACSS1 contributes to radioresistance—a critical barrier across solid tumours—remains completely unexplored.

In this study, we identified ACSS1 as a metabolic linchpin of radioresistance in breast cancer. ACSS1 was aberrantly upregulated in breast tumours and predicted reduced radiotherapy efficacy. Notably, IR induces metabolically coupled pyruvate-ROS flux that drives de novo acetate synthesis. ACSS1 upregulation amplifies this IR-triggered acetate production, thereby increasing nuclear acetyl-CoA accumulation, which spatially correlated with elevated histone acetylation and enhanced chromatin accessibility specifically at DNA damage loci. These changes promote HR repair, leading to radioresistance in breast cancer. By disrupting the mitochondrial-nuclear acetyl-CoA axis, ACSS1 inhibition offers a precision strategy to overcome HR-mediated resistance, addressing a critical unmet need in breast cancer radiotherapy.

## Results

### ACSS1 is identified as a candidate mediator of radiotherapy resistance in breast cancer

**Fig. 1:**
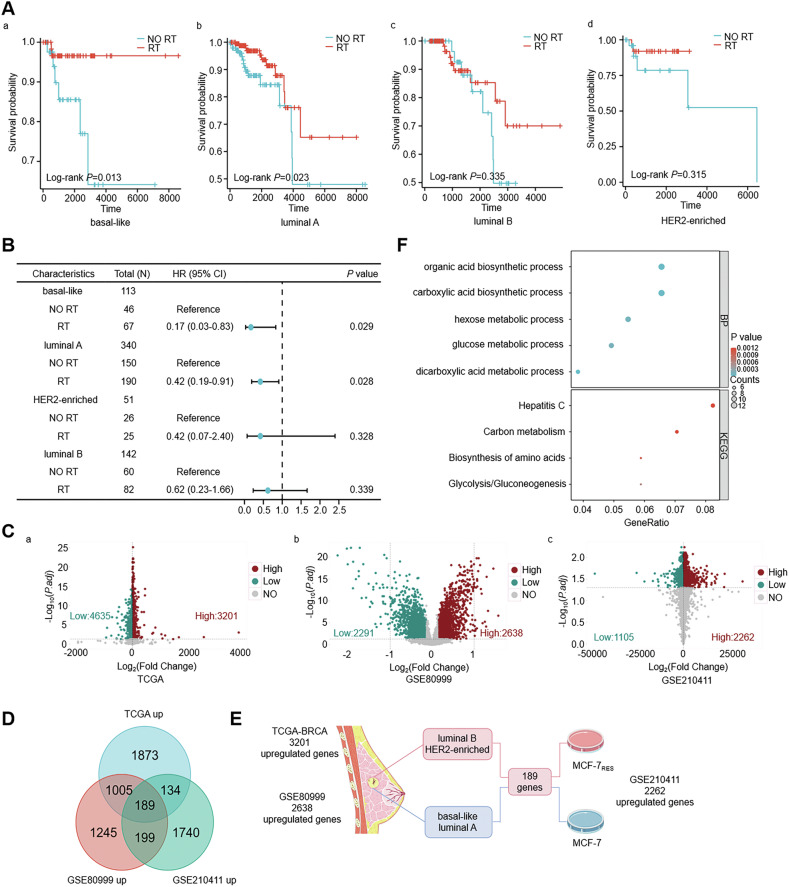
Identification of radioresistance-related genes through integrated analysis of TCGA and GEO databases. **A** Kaplan–Meier analysis of radiotherapy benefit in the TCGA cohort stratified by PAM50 subtypes: basal-like (a), luminal A (b), luminal B (c), and HER2-enriched (d). Log-rank test *P*-values are shown. **B** Forest plot showing subtype-specific associations with radiotherapy prognosis (hazard ratios ± 95% CI). **C** Volcano plots of DEGs in TCGA (a), GSE80999 (b), and GSE210411 (c) datasets. Red: upregulated genes; blue: downregulated genes (|Log_2_(Fold Change)| > 0.15, *P.adj* < 0.05). **D** Venn diagram identifying 189 consensus upregulated genes across TCGA and GEO datasets. **E** Schematic representation of the experimental workflow. **F** Functional enrichment of consensus DEGs highlights key biological processes (GO-BP) and signaling pathways (KEGG) associated with radioresistance.

Current evidence indicates variable radiotherapy benefits across breast cancer molecular subtypes. Using the TCGA database, we analyzed the survival impact of radiotherapy in breast cancer patients stratified by PAM50 subtypes [22] and randomized to treatment or non-radiotherapy cohorts (Table S1). Results showed that radiotherapy significantly improved overall survival in basal-like and luminal A patients, but no significant survival benefit was observed in luminal B or HER2-enriched subgroups (Fig. 1A). Multivariate Cox regression confirmed this subtype-specific benefit (Fig. 1B). To dissect molecular drivers of resistance, differential gene expression analysis between radiosensitive (basal-like + luminal A) and radioresistant (luminal B + HER2-enriched) groups identified 7836 differentially expressed genes (DEGs) in TCGA. Cross-validation with GEO datasets (GSE80999 and GSE210411) yielded 189 consistently upregulated genes in resistant subtypes (Fig. 1C–E). Pathway enrichment analysis implicated carbon metabolism, amino acid biosynthesis, and glycolysis/gluconeogenesis as key contributors to radioresistance (Fig. 1F). These findings highlight metabolic reprogramming as a central mechanism underlying radiotherapy resistance, offering novel targets for therapeutic intervention.

**Fig. 2:**
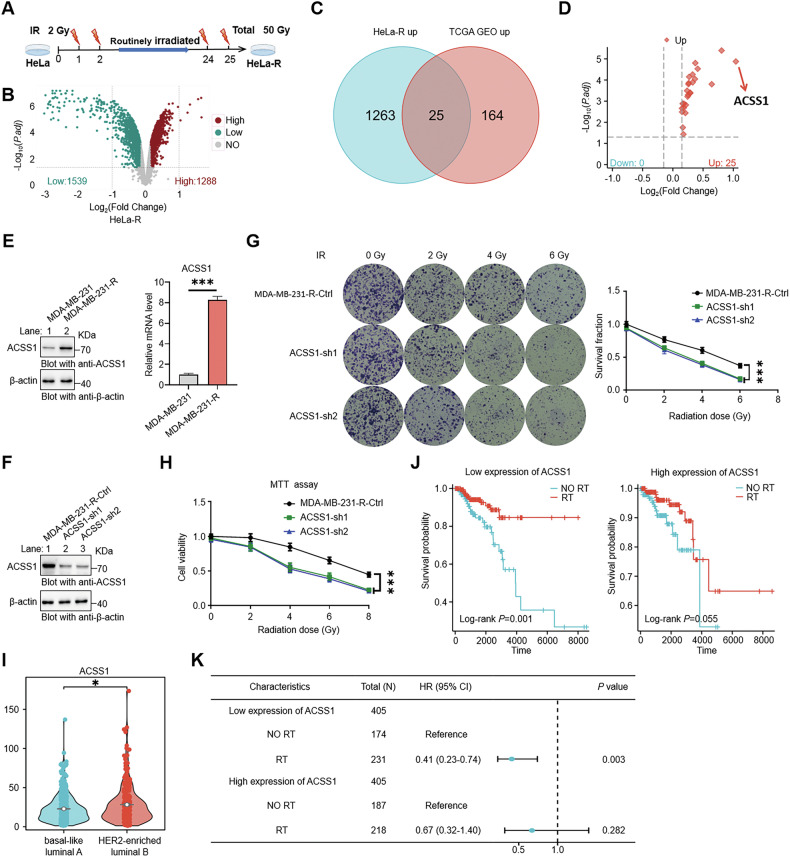
Generation of IR-resistant cell lines and identification of ACSS1. **A** Establishment of radioresistant HeLa-R cells through iterative irradiation. **B** Volcano plot of DEPs between parental and HeLa-R cells. Red: upregulated proteins; blue: downregulated proteins. **C** Venn diagram identifying 25 consensus upregulated genes overlapping DEPs and radioresistance-related DEGs. **D** Volcano plot of co-upregulated 25 DEPs. **E** ACSS1 expression in parental and MDA-MB-231-R cells. Protein levels were analyzed by western blot and mRNA levels by RT-qPCR. **F** Expression level of ACSS1 in MDA-MB-231-R cells. Colony formation (**G**) and MTT assay (**H**) of ACSS1-depleted MDA-MB-231-R cells exposed to the indicated doses of radiation. **I** Comparison of the mRNA expression of ACSS1 in radiosensitive group (basal-like + luminal A subtypes, *n* = 574) and radioresistant group (luminal B + HER2-enriched subtypes, *n* = 261) in the TCGA cohort. **J** Kaplan–Meier analysis of radiotherapy benefit in the TCGA cohort stratified by ACSS1 expression levels. **K** Forest plot depicting the correlation between ACSS1 expression levels and prognosis following radiotherapy. Each bar represents the mean ± SD for triplicate experiments. Statistical analysis was performed using two-tailed unpaired Student’s *t*-test for **E**; two-way ANOVA for **G** and **H**; Wilcoxon rank sum test for **I**; *P < 0.05; ***P < 0.001.

To identify molecular markers for radioresistance, we generated radiation-resistant HeLa cells (HeLa-R) through fractionated X-ray irradiation (2 Gy/fraction, 25 cycles) (Fig. 2A). HeLa-R cells exhibited robust radioresistance, with higher survival and proliferation post-IR compared to parental cells (Fig. S1A–C). TMT-based proteomics identified 2827 differentially expressed proteins (DEPs) in HeLa-R cells (Fig. 2B). RNA-seq and proteomics identified 25 genes upregulated at both mRNA and protein levels (Fig. 2C). We focused on the top-ranked protein ACSS1, pivotal in metabolism, with limited research on its radioresistance link (Fig. 2D). We observed that ACSS1 expression levels were significantly elevated in HeLa-R cells (Fig. S1D). ACSS1 knockdown in HeLa-R (ACSS1-sh1, ACSS1-sh2) reduced endogenous ACSS1 (Fig. S1E), decreasing cell survival, proliferation, and growth post-IR (Fig. S1F–H). To validate these findings in a breast cancer context, we generated radioresistant MDA-MB-231 cells (MDA-MB-231-R) using the same irradiation protocol (Fig. S1I, J). Consistent with the HeLa-R model, ACSS1 expression was significantly upregulated in MDA-MB-231-R cells (Fig. 2E). Moreover, knockdown of ACSS1 in MDA-MB-231-R cells similarly reduced cell survival and proliferation following irradiation (Fig. 2F–H). These results confirm that ACSS1 correlates strongly with radioresistance, and its deficiency heightens radiosensitivity in the radioresistant breast cancer cells.

Clinically, pan-cancer analysis confirmed the overexpression of ACSS1 in breast cancer (Fig. S2A). We evaluated the correlation between ACSS1 and clinical characteristics of breast cancer (Fig. S2B–H), detecting higher ACSS1 levels in the radioresistant (luminal B + HER2-enriched) group than in the radiosensitive (basal-like + luminal A) group (Fig. 2I). Clinical analysis showed that among patients with low ACSS1 expression, radiotherapy significantly prolonged overall survival; however, no significant survival benefit was observed in those with high ACSS1 expression (Fig. 2J). Multivariate Cox regression also confirmed this benefit (Fig. 2K). These clinical and prognostic associations prompted us to directly investigate whether ACSS1 functionally contributes to radiotherapy resistance in breast cancer.

### ACSS1 promotes radioresistance in breast cancer cells

**Fig. 3:**
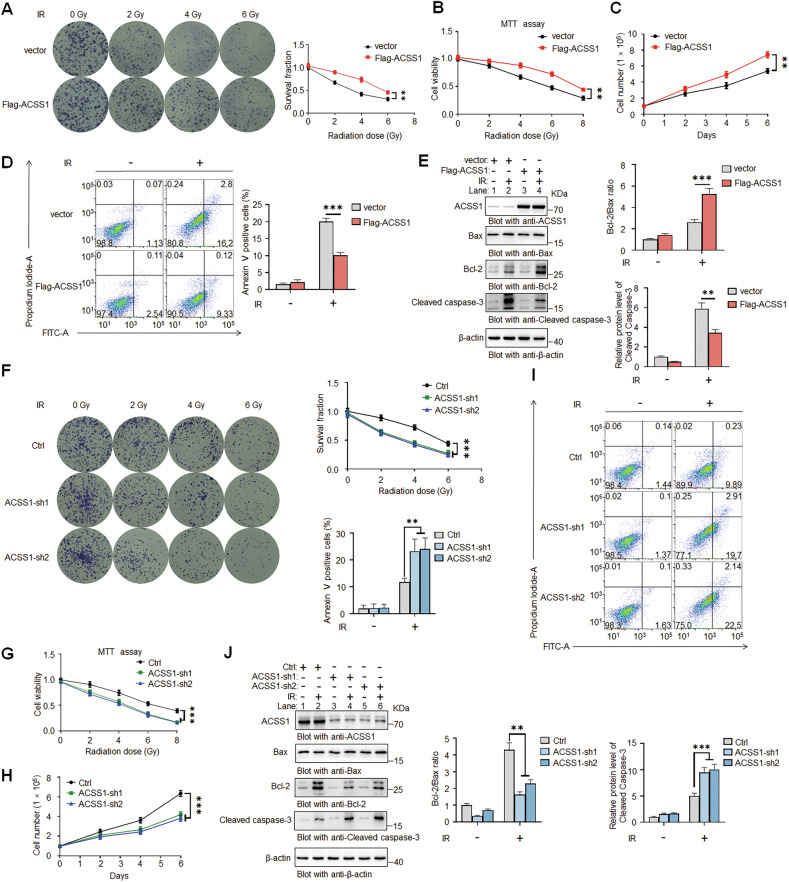
Modulation of ACSS1 expression controls radiosensitivity in breast cancer cells. Overexpression of ACSS1 increased the viability and proliferative potency of MDA-MB-231 cells treated with the indicated doses of radiation and analysed using colony formation (**A**) and MTT assay (**B**). **C** The cell growth viability was determined in MDA-MB-231 cells after 4 Gy IR. **D** Detection of apoptosis using Annexin V/PI staining in MDA-MB-231 cells after 10 Gy IR. **E** Western blot showing the apoptosis-related markers in MDA-MB-231 cells following 10 Gy IR. Depletion of ACSS1 decreases the viability and proliferative potency of BT474 cells treated with the indicated doses of radiation and analysed using colony formation (**F**) and MTT assay (**G**). **H** The cell growth viability was determined in BT474 cells after 4 Gy IR. **I** Detection of apoptosis using Annexin V/PI staining in BT474 cells after 10 Gy IR. **J** Western blot showing the apoptosis-related markers in BT474 cells following 10 Gy IR. Each bar represents the mean ± SD for triplicate experiments. Statistical analysis was performed using two-way ANOVA; **P < 0.01; ***P < 0.001.

To establish ACSS1’s functional role in breast cancer radioresistance, we employed bidirectional genetic models: ACSS1 overexpression in ACSS1-low MDA-MB-231 (basal-like) cells and knockdown (ACSS1-sh1, ACSS1-sh2) in ACSS1-high BT474 (luminal B) cells. Overexpression in MDA-MB-231 cells significantly enhanced clonogenic survival, viability, and cell growth (Fig. 3A–C) upon exposure to IR, while suppressing apoptosis as evidenced by reduced apoptosis rates (Fig. 3D), increased Bcl-2/Bax ratio, and attenuated caspase-3 cleavage (Fig. 3E). Conversely, ACSS1 silencing in BT474 cells markedly impaired cell survival, viability, and cell growth after IR treatment (Fig. 3F–H), while amplifying apoptosis through elevated apoptosis rates (Fig. 3I), decreased Bcl-2/Bax ratio, and robust caspase-3 activation (Fig. 3J). Collectively, these data support the conclusion that ACSS1 is a key determinant of breast cancer cell radiosensitivity, highlighting its potential as a target for enhancing breast cancer radiotherapy.

### ACSS1 facilitates the maintenance of genomic stability in response to DNA damage

**Fig. 4:**
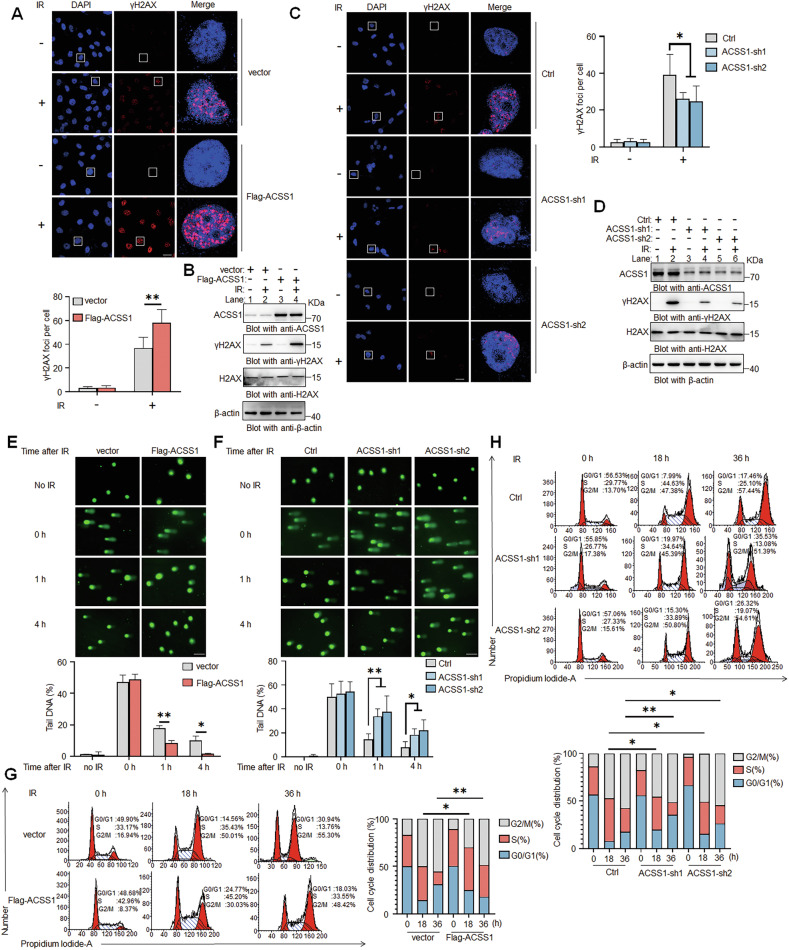
ACSS1 is a key regulator of genome stability. Immunofluorescence assay showing the number of γH2AX foci in MDA-MB-231 (**A**) and BT474 cells (**C**) 1 h after 10 Gy IR. Scale bar: 25 μm. Quantification of γH2AX foci number per nucleus in cells. Fifty nuclei were counted in each group. Western blot showing the expression of γH2AX in MDA-MB-231 (**B**) and BT474 cells (**D**) following 10 Gy IR treatment. The comet assay was employed to quantify comet tail lengths in MDA-MB-231 (**E**) and BT474 cells (**F**) after 10 Gy IR. Scale bar: 100 μm. Comet tail length was determined using CaspLab. At least 40 cells were analyzed in each sample. The cell cycle distribution was determined by flow cytometry in MDA-MB-231 (**G**) and BT474 cells (**H**) after 10 Gy treatment. The percentage in each phase is shown in the histogram. Each bar represents the mean ± SD for triplicate experiments. Statistical analysis was performed using two-way ANOVA; *P < 0.05; **P < 0.01.

Radiotherapy eradicates tumours by inducing DNA damage, yet cancer cells evade this through efficient repair mechanisms. To explore ACSS1’s role in this process, we utilized multiple approaches to assess phosphorylated histone H2AX (γH2AX), a critical DNA damage sensor that amplifies the damage signals and is vital for preserving genomic integrity [23, 24]. In ACSS1-overexpressing MDA-MB-231 cells, IR triggered amplified γH2AX signaling, evidenced by increased foci formation (Fig. 4A) and elevated protein levels (Fig. 4B), indicating enhanced damage sensing. Conversely, ACSS1-silenced BT474 cells exhibited diminished γH2AX foci (Fig. 4C) and expression (Fig. 4D). Functional comet assays revealed accelerated DNA repair in ACSS1-high cells (shorter tail lengths; Fig. 4E) versus impaired repair in ACSS1-low cells (prolonged tails; Fig. 4F), directly linking ACSS1 to repair proficiency. Additionally, cell cycle analysis demonstrated that ACSS1-overexpressing cells had significant S phase arrest following DNA damage (Fig. 4G), providing temporal windows for repair machinery activation, while ACSS1 depletion disrupted this arrest (Fig. 4H), compromising damage resolution. Collectively, these findings strongly support the notion that ACSS1 plays pivotal roles in DNA repair mechanisms and enhances genomic stability in breast cancer cells in response to DNA damage.

### ACSS1 coordinates acetyl-CoA dynamics to enable histone acetylation during genotoxic stress

**Fig. 5:**
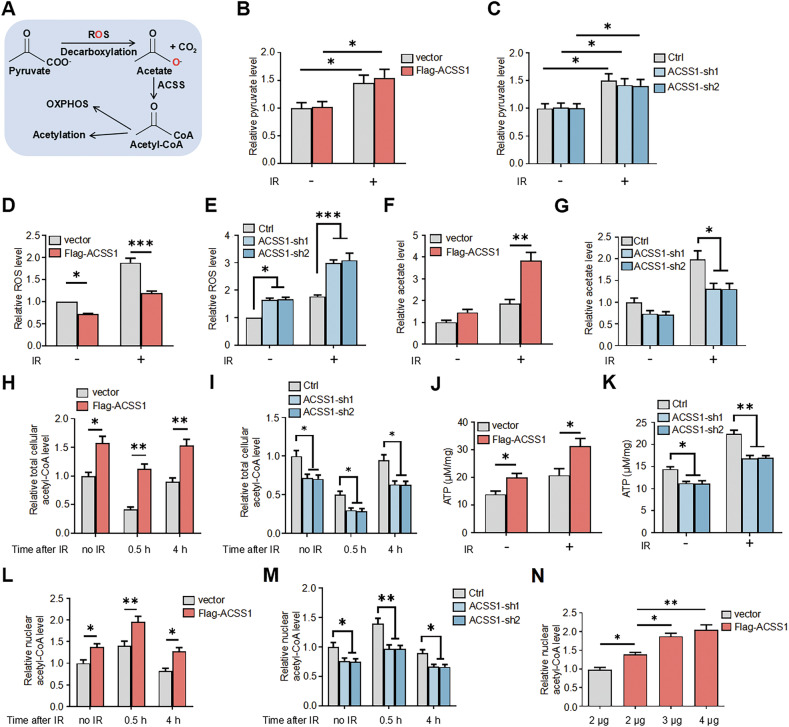
ACSS1 regulates acetyl-CoA homeostasis and bioenergetics in response to IR. **A** Schematic representation of ROS-mediated pyruvate oxidative decarboxylation to acetate. Pyruvate levels in MDA-MB-231 (**B**) and BT474 (**C**) cells were examined 4 h after 10 Gy IR. Intracellular ROS levels in MDA-MB-231 (**D**) and BT474 (**E**) cells were examined following IR treatment. Acetate levels in MDA-MB-231 (**F**) and BT474 (**G**) cells were examined 4 h after 10 Gy IR. Quantifications of total cellular acetyl-CoA levels from MDA-MB-231 (**H**) and BT474 (**I**) cells treated in the indicated conditions following IR treatment. ATP levels in MDA-MB-231 (**J**) and BT474 (**K**) cells were examined after IR treatment. Quantifications of nuclear acetyl-CoA levels from MDA-MB-231 (**L**) and BT474 (**M**) cells treated in the indicated conditions following IR treatment. **N** Nuclear acetyl-CoA levels were quantified 4 h after 10 Gy IR in cells transfected with exogenous ACSS1 at varying concentrations. Each bar represents the mean ± SD for triplicate experiments. Statistical analysis was performed using two-way ANOVA for **B**–**M**; One-way ANOVA for **N**; *P < 0.05; **P < 0.01; ***P < 0.001.

Integrated analysis of TCGA and GEO datasets established metabolic reprogramming as a hallmark of radioresistant tumours. Intriguingly, our results revealed that IR triggers a metabolic cascade marked by pyruvate accumulation and ROS elevation in cells (Fig. 5A–E), consistent with mechanisms of de novo acetate synthesis through pyruvate-ROS coupling [25]. ACSS1-overexpressing cells showed significantly attenuated ROS generation compared to vector controls—a phenotype reversed upon ACSS1 knockdown (Fig. 5D, E). These data suggest that ACSS1 activity accelerates ROS-coupled acetate synthesis, thereby reducing oxidative stress while driving acetate production. This mechanism aligns with our observation that IR induces an elevation of cellular acetate levels, an effect further amplified by ACSS1 overexpression (Fig. 5F) and conversely attenuated upon ACSS1 knockdown (Fig. 5G). We next asked whether the ROS-reducing effect of ACSS1 depended on the availability of endogenous acetate precursors. While ACSS1 overexpression reduced ROS levels both with and without IR in standard medium, this advantage persisted in the absence of IR but was markedly attenuated following IR upon supplementation with sodium acetate (NaAc) (Fig. S3A). Specifically, NaAc treatment elevated ROS and diminished the differential effect of ACSS1 overexpression after IR, indicating that it sustains high ROS levels by saturating the ACSS1-mediated ROS consumption pathway.

We therefore investigated how ACSS1-mediated acetate metabolism regulates acetyl-CoA dynamics during radiation response. Time-course analysis revealed dynamic IR-induced fluctuations in acetyl-CoA pools, characterized by a transient 30-min depletion phase followed by recovery within 4 h. ACSS1 overexpression amplified total cellular acetyl-CoA levels across all phases (Fig. 5H), while its knockdown exacerbated depletion and impaired recovery (Fig. 5I), underscoring ACSS1’s critical role in acetyl-CoA homeostasis during IR stress. Notably, total cellular acetyl-CoA measurements primarily reflected mitochondrial reserves [26], given the compartmentalized nature of acetyl-CoA metabolism in mammalian cells. Immunofluorescence analysis further confirmed that Flag-tagged ACSS1 colocalized with the mitochondrial marker COXIV, indicating its mitochondrial localization (Fig. S3B). Functionally, ACSS1 overexpression promoted ATP generation (Fig. 5J), whereas knockdown suppressed it (Fig. 5K), linking metabolic flux to bioenergetic adaptation. To delineate compartment-specific dynamics, nuclear fractionation analysis revealed divergent temporal patterns: vector controls exhibited rapid but transient nuclear acetyl-CoA accumulation post-IR, while ACSS1-overexpressing cells showed amplified early-phase nuclear enrichment with delayed resolution (Fig. 5L). Conversely, ACSS1 knockdown diminished nuclear acetyl-CoA accumulation (Fig. 5M), and this regulation occurred in a dose-dependent manner (Fig. 5N). Notably, inhibition of the citrate transporter (CTPI-2) or ACLY (SB 204990) suppressed the ACSS1-dependent increase in nuclear acetyl-CoA (Fig. S3C). This pharmacological evidence suggests that the ACSS1-dependent generation of nuclear acetyl-CoA is mechanistically reliant on the citrate-ACLY axis. These findings position ACSS1 as an IR-responsive metabolic coordinator that spatiotemporally integrates pyruvate-ROS-acetate flux with mitochondrial-nuclear acetyl-CoA partitioning to fuel irradiation-induced biosynthetic demands.

**Fig. 6:**
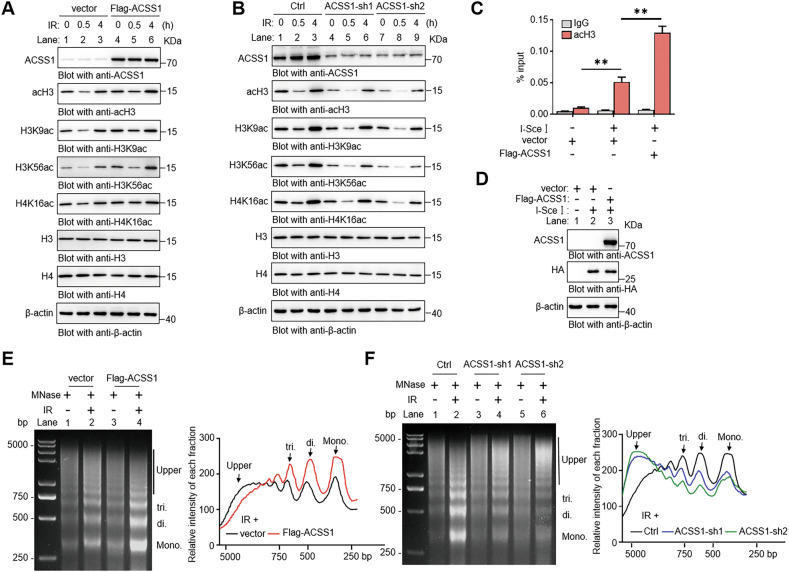
ACSS1-dependent nuclear acetyl-CoA promotes histone acetylation and chromatin relaxation at damage sites. Western blot showing specific antibody signals for acH3, H3K9ac, H3K56ac and H4K16ac from MDA-MB-231 (**A**) and BT474 (**B**) cells. **C** ChIP was performed with anti-acH3 or anti-IgG as a negative control in U2OS-DR-GFP cells transfected with control vector or I-SceI. **D** Western blot of HA-I-SceI. MNase sensitivity assay of chromatin condensation was performed in MDA-MB-231 (**E**) and BT474 cells (**F**) following 10 Gy IR treatment. The same amount of DNA (2 μg) was subjected to agarose gel, and the density of each nucleosome was calculated by ImageJ. Each bar represents the mean ± SD for triplicate experiments. Statistical analysis was performed using two-way ANOVA; **P < 0.01.

Nuclear acetyl-CoA serves as the essential substrate for histone acetylation, a process critical for DNA damage response (DDR). We demonstrate that ACSS1 governs this metabolic-epigenetic axis under genotoxic stress. ACSS1 overexpression robustly enhanced both global histone H3 acetylation (acH3) and DDR-linked site-specific modifications—including H3K9ac, H3K56ac, and H4K16ac—under genotoxic stress [27–29], while ACSS1 knockdown conversely diminished these epigenetic marks (Fig. 6A, B). To exclude a potential role of the acetyl-CoA synthetase ACSS2, we treated cells with its inhibitor VY-3-135. While ACSS2 inhibition reduced overall nuclear acetyl-CoA levels, it did not abolish the ACSS1-overexpression phenotype (Fig. S3D). The persistence of ACSS1’s function under these conditions underscores its non-redundant and compartment-specific role in maintaining the nuclear acetyl-CoA pool during genotoxic stress. To dissect ACSS1’s role in a spatially controlled damage model, we utilized a U2OS-DR-GFP reporter system, where I-SceI endonuclease-induced DSBs enabled precision analysis of localized chromatin events. The chromatin immunoprecipitation (ChIP) analysis revealed ACSS1-dependent acH3 enrichment at DSB sites (Fig. 6C), with I-SceI expression confirmed by immunoblotting (Fig. 6D). Given negligible endogenous ACSS1 in U2OS cells (Fig. S3E), overexpression studies were prioritized. Functional validation via MNase sensitivity assays showed that ACSS1 potentiates IR-induced chromatin decompaction, evidenced by elevated mono-/di-/tri-nucleosome density (Fig. 6E). Strikingly, ACSS1 knockdown conversely reduced chromatin accessibility in BT474 cells (Fig. 6F). Collectively, these findings support the idea that ACSS1 might participate in maintaining acetyl-CoA pools to enable proper histone acetylation dynamics and facilitate chromatin relaxation in response to DNA damage.

### ACSS1 promotes HR-mediated DSB repair

Our data thus far demonstrate that ACSS1 amplifies the nuclear acetyl-CoA pool to fuel histone acetylation and chromatin relaxation. We sought to determine whether this metabolic priming function autonomously triggers the DDR or specifically facilitates repair upon damage infliction. To test this, we supplemented unirradiated vector and ACSS1-overexpressing cells with acetate, using IR-treated vector cells as a positive control for DDR activation. Notably, acetate supplementation affected neither γH2AX protein levels nor the formation of γH2AX or BRCA1 foci in the absence of IR (Fig. S4A, B). This key finding indicates that ACSS1-mediated metabolic reprogramming prepares the chromatin landscape but requires a genuine DNA damage signal to engage the DDR machinery. Together, these data delineate a two-step mechanism: ACSS1-mediated metabolic priming creates a permissive chromatin state, which is then engaged by the DNA damage signal to efficiently recruit the DNA repair machinery.

**Fig. 7:**
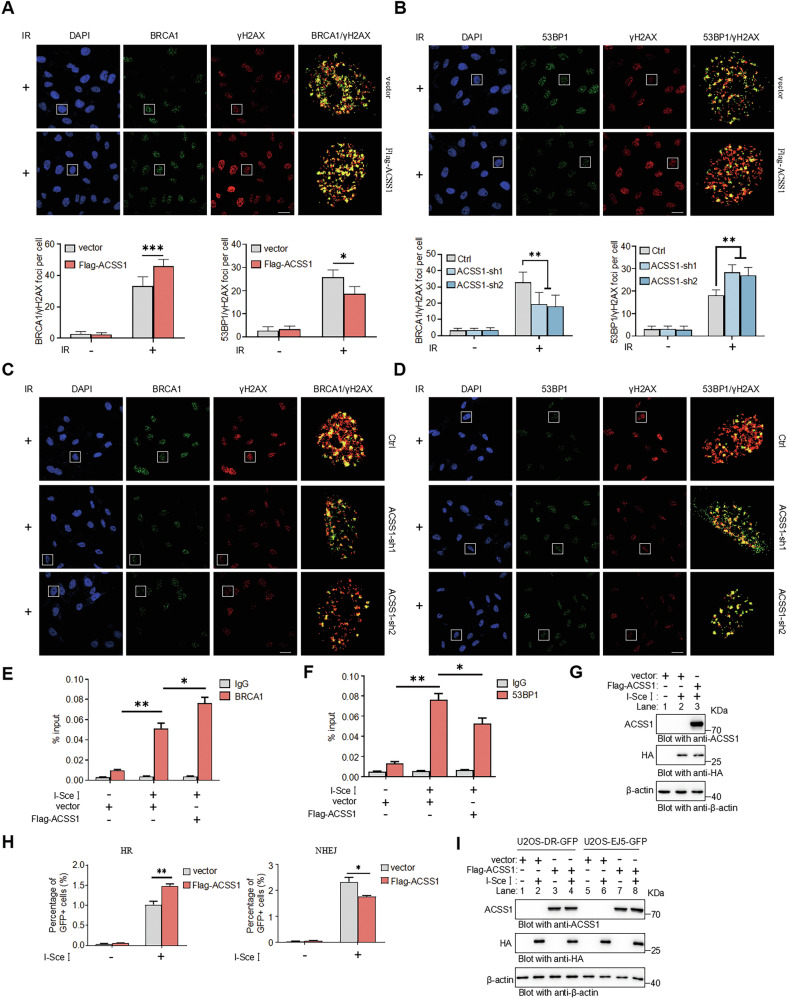
ACSS1 is required for efficient HR repair. Immunofluorescence assay showing the number of BRCA1 foci in MDA-MB-231 (**A**) and BT474 cells (**C**) 1 h after 10 Gy IR. Scale bar: 25 μm. Quantification of BRCA1 foci number per nucleus in cells. Fifty nuclei were counted in each group. Immunofluorescence assay showing the number of 53BP1 foci in MDA-MB-231 (**B**) cells and BT474 cells (**D**) 1 h after 10 Gy IR. Scale bar: 25 μm. Quantification of 53BP1 foci number per nucleus in cells. Fifty nuclei were counted in each group. ChIP of BRCA1 (**E**) and 53BP1 (**F**) in U2OS-DR-GFP cells with or without I-SceI transfection. **G** Western blot of ACSS1 and HA-I-SceI. **H** HR and NHEJ repair efficiencies were determined in U2OS-DR-GFP and U2OS-EJ5-GFP reporter cells. **I** Western blot of ACSS1 and HA-I-SceI. Each bar represents the mean ± SD for triplicate experiments. Statistical analysis was performed using two-way ANOVA for **A**–**F** and **H**; *P < 0.05; **P < 0.01; ***P < 0.001.

We therefore hypothesized that ACSS1 functions to enhance the efficiency and fidelity of DNA repair pathways specifically upon genotoxic stress. Recent studies have emphasized the crucial role of dynamic histone acetylation in recruiting DNA repair proteins and determining the choice of DNA repair pathways, particularly HR and NHEJ, in response to DSBs [30–32]. Given this, we aimed to explore the potential role of ACSS1 within the DSB repair pathway. Initial immunofluorescence analyses revealed that ACSS1 overexpression preferentially enhanced BRCA1 foci formation while suppressing 53BP1 accumulation at γH2AX-marked DNA damage sites (Fig. 7A, B, Fig. S5A, B), whereas ACSS1 depletion elicited opposite effects (Figs. 7C, D, and S5C, D). Subsequent ChIP analysis in U2OS-DR-GFP cells revealed that ACSS1 overexpression enhanced BRCA1 occupancy while reducing 53BP1 binding at engineered DNA break loci (Fig. 7E, F). To functionally link these recruitment patterns to repair outcomes, we quantified HR and NHEJ efficiencies using I-SceI-induced DSB repair assays. ACSS1 overexpression robustly enhanced HR proficiency while attenuating NHEJ activity (Figs. 7H and S5E), a bias mechanistically aligned with its S-phase regulatory function. Western blot verified ACSS1 and I-SceI expression (Fig. 7G, I). Collectively, these results indicate that ACSS1 participates in histone acetylation dynamics in response to DNA damage, thereby promoting error-free HR-mediated DNA repair.

### ACSS1 confers radiotherapy resistance in breast cancer cells

**Fig. 8:**
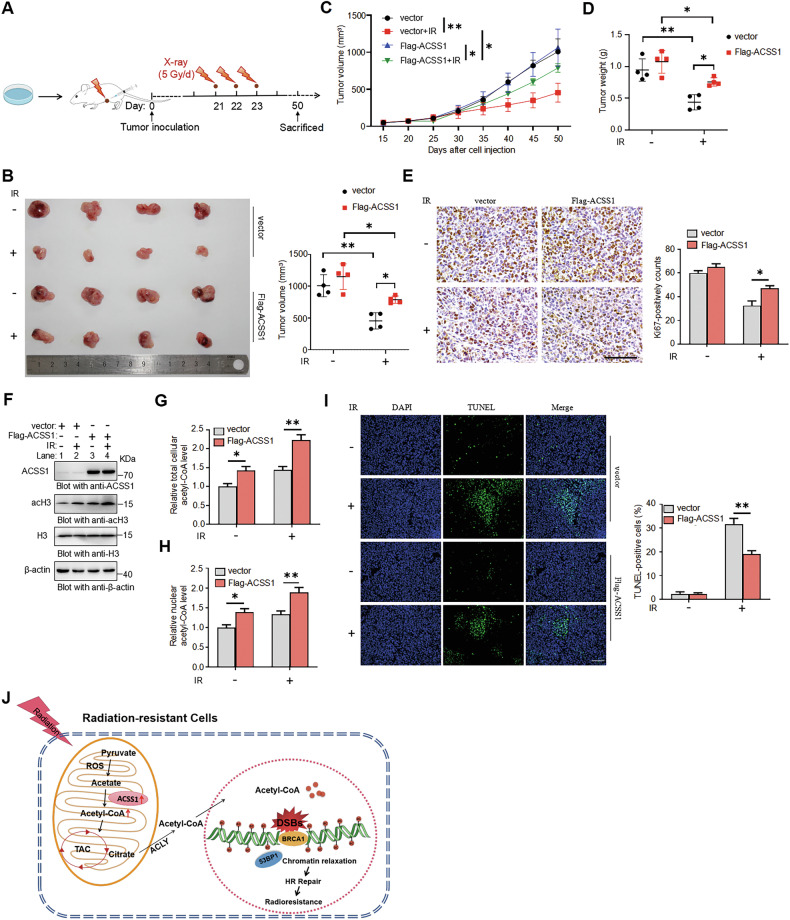
ACSS1 reduces the sensitivity of breast cancer cells to radiotherapy. **A** Schematic representation of the experimental design in mouse model. The breast cancer xenograft model was established using MDA-MB-231 cells stably expressing vector and Flag-ACSS1. **B** Representative images of xenografts from different treatment groups. Tumour growth curves (**C**) and endpoint tumour weights at sacrifice (**D**) of xenografts from different treatment groups (*n* = 4 mice per group). **E** Immunohistochemistry analyses of breast tumour tissues with the antibody against Ki67. Scale bar: 100 μm. **F** Western blots of xenograft with the antibody against acH3. Quantifications of total cellular (**G**) and nuclear (**H**) acetyl-CoA levels. **I** TUNEL assay to assess apoptotic cells in xenografts. Scale bar: 100 μm. J Working model of the role of ACSS1 in the radioresistance. Each bar represents the mean ± SD for triplicate experiments. Statistical analysis was performed using two-way ANOVA; *P < 0.05; **P < 0.01.

To evaluate the clinical implications of ACSS1 in breast cancer radioresistance, we established orthotopic xenografts using MDA-MB-231 cells with or without ACSS1 overexpression in nude mice (Fig. 8A). Following localized irradiation, ACSS1-overexpressing tumours exhibited accelerated growth kinetics and increased mass compared to controls, indicating reduced radiation sensitivity (Fig. 8B–D). This radioresistance phenotype correlated with elevated proliferative activity, as evidenced by enhanced Ki67 staining in ACSS1-overexpressing tumours (Fig. 8E). Meanwhile, irradiated ACSS1-high tumours showed marked increases in global acetylation of histone H3 (Fig. 8F). Furthermore, ACSS1 amplification significantly boosted both total and nuclear acetyl-CoA pools in tumour tissues, establishing a direct metabolic link to its epigenetic effects (Fig. 8G, H). Importantly, TUNEL assays confirmed diminished apoptosis in ACSS1-overexpressing tumours post-IR, reinforcing its functional role in cell survival under genotoxic stress (Fig. 8I). Collectively, these preclinical findings position ACSS1 as a critical modulator of radiotherapy response in breast cancer, highlighting the therapeutic potential of disrupting its acetyl-CoA metabolic axis to overcome radioresistance.

## Discussion

Mitochondrial ACSS1 is established herein as a central regulator of radioresistance in breast cancer, orchestrating a metabolic-epigenetic axis that enhances HR-mediated repair (Fig. 8J). While the importance of nuclear acetyl-CoA for histone acetylation and DNA repair has been established [16], the critical metabolic sources supplying this nuclear pool under genotoxic stress have remained less clear. Our work positions ACSS1 as a pivotal driver that converts radiation-induced mitochondrial stress into a citrate-dependent acetyl-CoA flux to the nucleus, thereby directly challenging the conventional model of strictly compartmentalized acetyl-CoA metabolism. Notably, our functional studies demonstrate a causal role for ACSS1 in IR-specific radioresistance: its overexpression significantly reduces radiation sensitivity specifically under IR stress, while its knockdown increases sensitivity, without acting as a general oncogenic driver in unirradiated conditions. ACSS1 orchestrates radioresistance by metabolically amplifying the DNA damage response. It operates through a damage-responsive mechanism: after establishing a primed chromatin state, it harnesses IR-triggered acetate surge to fuel histone acetylation and HR repair, converting metabolic stress into a repair advantage. This makes ACSS1 a promising therapeutic target for sensitizing HR-proficient breast cancers to radiotherapy.

Our study demonstrates that ACSS1-generated mitochondrial acetyl-CoA orchestrates nuclear chromatin remodeling at radiation-induced DNA breaks through a citrate-dependent interorganellar metabolite shuttle. This model provides direct experimental evidence addressing the long-standing question of mitochondrial-nuclear acetyl-CoA coupling [33], positioning ACSS1-citrate-ACLY as an important pathway in this context. While alternative mechanisms—such as direct acetyl-CoA transport [34] or non-canonical pyruvate dehydrogenase complex activity [35]—cannot be fully excluded, our inhibitor studies confirm its central role in facilitating ACSS1-mediated metabolic-epigenetic crosstalk. Furthermore, the failure of ACSS2 inhibition to attenuate ACSS1-driven nuclear acetyl-CoA enrichment confirms the functional segregation of mitochondrial ACSS1 from ACSS2, whereas ACSS2 primarily fuels lipid synthesis under hypoxia [36]. This demonstrates its non-redundant, compartment-specific role in genotoxic stress and reveals how tumours exploit distinct enzymatic isoforms for different environmental challenges. We thus reveal that radiation-induced mitochondrial stress amplifies acetyl-CoA flux via ACSS1 to drive Tip60-dependent H4K16 acetylation, displacing 53BP1, enhancing BRCA1 recruitment, and promoting HR repair [32, 37]—a process previously attributed largely to nuclear acetyl-CoA pools. Crucially, ACSS1 depletion outperforms ACLY inhibition by simultaneously starving chromatin plasticity through nuclear acetyl-CoA loss [38] and disrupting mitochondrial energy adaptation via acetate catabolism blockade, positioning ACSS1 as a dual metabolic-epigenetic vulnerability.

Therapeutic exploitation of this axis requires confronting its spatial complexity. The mitochondrial localization of ACSS1 necessitates targeted inhibitor delivery strategies, such as triphenylphosphonium-conjugated agents [39, 40], to overcome dual membrane barriers. Our in vivo studies using MDA-MB-231 xenografts demonstrated that ACSS1 overexpression drives radioresistance, with ACSS1-high tumours exhibiting significantly attenuated radiation response compared to vector controls. Subtype-specific analysis further revealed that luminal B/HER2-enriched breast cancers, which frequently exhibit endogenous ACSS1 upregulation, show heightened radioresistance, suggesting these tumours are prime candidates for ACSS1-targeted therapy. In contrast, basal-like/luminal A subtypes, characterized by low ACSS1 expression, likely employ resistance mechanisms independent of acetyl-CoA-mediated chromatin remodeling, underscoring the need for molecular stratification. To address potential compensatory pathways such as SMCT1-dependent acetate uptake [41], combining ACSS1 inhibitors with acetate-depleting therapies or HDAC inhibitors may enforce sustained chromatin compaction. Clinically, ¹¹C-acetate PET imaging [42, 43] could non-invasively identify tumours reliant on this ACSS1-mediated metabolic axis, enabling patient stratification for such metabolic radiosensitization strategies. This approach exploits ACSS1’s dual role as a metabolic and epigenetic regulator, thereby converting its overexpression—a marker of poor prognosis—into a therapeutic vulnerability through targeted metabolic disruption and advancing precision radiotherapy beyond anatomic parameters.

The upregulation of ACSS1 in radiotherapy-refractory breast malignancies may arise through adaptive transcriptional and metabolic reprogramming under therapeutic stress. Radiation-induced hypoxia and oxidative stress may activate stress-responsive transcription factors, including KLF15—a known inducer of ACSS1 during nutrient deprivation [44]—and hypoxia [19]-inducible factors, including HIF-1α, potentially synergizing to upregulate ACSS1. Concurrently, radiotherapy may elevate extracellular acetate availability, as supported by our data showing radiation-triggered intracellular acetate accumulation and ACSS1-enhanced utilization. Mitochondrial ACSS1 is thus uniquely positioned to exploit acetate as an alternative carbon source, sustaining acetyl-CoA pools for both ATP production and nuclear histone acetylation—a process critical for DNA repair. This metabolic adaptation links mitochondrial resilience directly to chromatin plasticity, providing a cohesive survival strategy under genotoxic stress. While these findings suggest ACSS1 as a mediator of metabolic-epigenetic crosstalk in radioresistance, further studies are needed to clarify KLF15/HIF-1α regulation and mitochondrial-nuclear acetyl-CoA dynamics. Therapeutic strategies targeting ACSS1 or acetate flux could disrupt this adaptive axis, though preclinical validation is essential to assess specificity and translational potential.

In conclusion, this study illuminates ACSS1 as a metabolic linchpin coordinating energy production, chromatin dynamics, and DNA repair fidelity under radiation stress. By bridging the gap between tumour metabolism and radiobiology, our findings advocate for a paradigm shift in overcoming radioresistance—one that targets the metabolic infrastructure enabling genomic adaptation rather than solely focusing on DNA damage itself.

## Materials and methods

### Cell culture, transfection and treatment

MDA-MB-231 and BT474 cell lines were obtained from the American Type Culture Collection (ATCC; Manassas, VA, USA) and cultured according to the manufacturer’s protocol. U2OS cells harboring either the DR-GFP or EJ5-GFP reporter systems were generously provided by Prof. Lei Shi. MDA-MB-231 and U2OS cells were maintained in Dulbecco’s Modified Eagle Medium (DMEM; Biological Industries, 06-1055-57-1 A) supplemented with 10% fetal bovine serum (FBS; Biological Industries, 04-001-1ACS), while BT474 cells were cultured in RPMI-1640 medium (Biological Industries, 01-100-1 A) containing 20% FBS. All cells underwent verification through the examination of their morphology and growth patterns, confirming their absence of mycoplasma contamination. Plasmid transfections were performed using Lipofectamine 2000 reagent (Invitrogen; 11668019) following the manufacturer’s optimized protocol. For exogenous acetate treatment, cells were incubated with 5 mM NaAc (MedChem Express, HY-Y1325H) for 24 h. Cells were treated with DMSO (vehicle), 60 µM CTPI-2 (MedChem Express, HY-123986), 60 µM SB 204990 (MedChem Express, HY-16450), and 10 µM VY-3-135 (MedChem Express, HY-145953) for 24 h, respectively, for acetyl-CoA quantification.

### X-ray irradiation and establishment of radioresistant cell lines

Radiation exposure was delivered using an X-ray generator (RS2000 PRO, Radsource Corporation; 160 kV, 25 mA). HeLa and MDA-MB-231 cells were cultured to 50% confluence and then subjected to 2 Gy of radiation. Upon achieving 90% confluence, the cells underwent trypsinization followed by subculturing. This fractionated radiation protocol was repeated until cumulative dose reached 50 Gy over 25 cycles, generating the radioresistant sublines designated HeLa-R and MDA-MB-231-R.

### Plasmids, lentivirus, and stable cell construction

The lentiviral plasmids pLVX-IRES-Flag-ACSS1 (overexpression vector), pLKO.1-ACSS1-sh1 (shRNA sequence: 5′-GGCCTACCCAGGCTATTACTT-3′) and pLKO.1-ACSS1-sh2 (5′-ATCACCTACAGGGAACTACTG-3′) were custom-synthesized by GenePharma (Shanghai, China). Lentivirus transfected stable cells were constructed as previously described [45].

### Western blot and antibodies

Cellular lysates were separated on 12% SDS-PAGE and immunoblotted by using specific antibodies: ACSS1 (Proteintech, #17138-1-AP; 1:1000 western blot), β-actin (Sigma-Aldrich; #A5441, 1:1000 western blot), Bax (Proteintech, #50599-2-Ig; 1:1 000 western blot), Bcl-2 (Proteintech, #26593-1-AP; 1:1500 western blot), cleaved caspase-3 (CST, #9664; 1:1000 western blot), γH2AX (phospho S139) (Abcam, #ab2893; 1:200 immunofluorescence, 1:1000 western blot), H2AX (Proteintech, #10856-1-AP; 1:1000 western blot), Histone H3 (Abcam, #ab10799; 1:1000 western blot), Histone H4 (Proteintech, #16047-1-AP; 1:1000 western blot), acetyl-Histone H3 (Millipore, #06-599; ChIP and 1:20,000 western blot), Histone H3K9ac (Abcam, #ab10812; 1:500 western blot); Histone H3K56ac (Proteintech, #39282; 1:2500 western blot), Histone H4K16ac (Abcam, #ab109463; 1:1000 western blot), BRCA1 (Proteintech, #22362-1-AP; ChIP and 1:1000 immunofluorescence), 53BP1 (Novus Biologicals, #NB100-304; ChIP and 1:2000 immunofluorescence); HA (Proteintech, #51064-2-AP; 1:5000 western blot), Ki67 (Proteintech, #27309-1-AP; 1:4000 immunohistochemistry).

### RNA extraction and RT-qPCR

Total RNA was isolated from cells using the TRIzol (Vazyme, #R401-01). Then, RNA was reverse transcribed into cDNA using a reverse transcription kit (Genstar, #A230-10). RT-qPCR was performed using 2× RealStar Fast SYBR qPCR Mix (Genstar, #A303-10). The results were normalized to *β-actin* levels. The primer sequences using for RT-qPCR were as follows: *ACSS1* forward, 5′-CGTCCTTTTTGAGAGCACCC-3′, reverse, 5′-GCATCACCGTATTTCAGCAACA-3′; *β-actin* forward, 5′-CACCATTGGCAATGAGCGGTTC-3′, reverse, 5′-AGGTCTTTGCGGATGTCCACGT-3′.

### Apoptosis assay

Cell apoptosis was analyzed using a flow cytometry-based Annexin V-FITC/PI staining protocol. After IR treatment, cells were harvested at specific time points, washed with PBS, and resuspended in staining buffer to a density of 1 × 10^5^ cells per sample. The cell suspension was incubated with Annexin V-FITC and PI in the dark for 15 min. Flow cytometry was immediately performed to quantify the proportion of Annexin V-FITC-positive cells, representing early and late apoptotic cells.

### MTT assay

Cell viability was assessed using methylthiazoletetrazolium (MTT) assays. Cells were seeded in 96-well culture plates at a density of 2000 cells/well and treated with various doses of IR. After a 3-day incubation period, 20 μL of MTT reagent (Sigma, 5 mg/mL) was added to each well and incubated at 37 °C for 4 h. Formazan crystals formed during the reaction were dissolved by adding DMSO. The absorbance of the resulting solution was then measured at a wavelength of 570 nm using spectrophotometry.

### Colony formation assay

Cell survival was assessed using a colony formation assay. Cells were seeded in 3.5-cm dishes and treated with varying doses of IR. After a 10-day incubation period, cells were washed with PBS, fixed in 4% paraformaldehyde for 15 min, and stained with crystal violet for 10 min. Following rinsing with cold PBS, the number of colonies was quantified.

#### Immunofluorescence

Cells were cultured on glass coverslips and subsequently fixed with 4% paraformaldehyde for 10 min at room temperature. Fixed samples were permeabilized using 0.2% Triton X-100 in PBS for 10 min, followed by blocking with 5% BSA in PBS for 1 h. Primary antibody incubation was performed at 4 °C overnight, followed by repeated washing. Fluorophore-conjugated secondary antibodies were incubated for 7 h at 4 °C. Imaging was performed using an Olympus FV1000 confocal microscope.

### Comet assay

DNA damage was assessed using the comet assay [46] at 0, 1, and 4 h post-IR with a reagent kit (Bio-Techne, #4250-050-K). Briefly, cells were harvested and resuspended in PBS at a density of 1 × 10^5^ cells/mL. Then the cell suspension was embedded in Comet LMAgarose and layered onto CometSlide^TM^. After gelation at 4 °C, the slides were immersed in ice-cold lysis solution for 1 h. The slides were incubated in a freshly prepared alkaline unwinding solution (200 mM NaOH, 1 mM EDTA, pH >13) for 1 h at 4 °C in the dark, followed by electrophoresis in electrophoresis buffer. After electrophoresis, slides were rinsed with deionized water, dehydrated in 70% ethanol, air-dried, and stained with SYBR Green Ⅰ (Solarbio, #SY1020) for 30 min at room temperature in the dark. Comet tail length was quantified using a fluorescence microscope and analyzed with CaspLab.

### Cell cycle analysis

For cell cycle analysis, cells were irradiated as indicated, harvested via centrifugation, and washed with PBS. Fixed in ice-cold 70% ethanol at –20 °C overnight to preserve cellular architecture and permeabilize membranes for DNA staining, cells were subsequently incubated with propidium iodide (PI) at 37 °C for 20 min in the dark. DNA content quantification was performed by flow cytometry.

### Pyruvate quantification

Pyruvate levels were measured using the Pyruvate Content Assay Kit (Solarbio, #BC2200) according to the manufacturer’s instructions. Briefly, 5 × 10⁶ cells were collected and lysed in extraction buffer. Following sonication, samples were left to stand for 30 min. After centrifugation at 8000 × *g* for 10 min at room temperature, the supernatant was collected, and the pyruvate levels were quantified by absorbance at 520 nm.

### Measurement of ROS

ROS levels were quantified using the ROS Assay Kit (Beyotime Biotechnology, #S0033S) according to the manufacturer’s instructions. Briefly, cells were collected and incubated with 10 µM DCFH-DA at 37 °C for 20 min. Following incubation, cells were washed three times with serum-free cell culture medium to remove the extracellular probe and resuspended in serum-free cell culture medium for analysis. The DCF fluorescence was measured by flow cytometry, and the mean fluorescence intensity was used to represent the intracellular ROS levels.

### Acetate assay

Acetate levels were quantified using the Acetate Assay Kit (Abcam, #ab204719) according to the manufacturer’s protocol. Briefly, cell lysates were added to a 96-well plate alongside acetate standards. The reaction mix, containing Acetate Assay Buffer, Enzyme Mix, Substrate Mix, ATP II, and Developer Solution III, was added to each well. After 40 min of incubation at room temperature, absorbance was measured at 450 nm.

### Acetyl-CoA quantification

Acetyl-CoA levels were quantified using the Acetyl-CoA Assay Kit (Solarbio, #BC0980) according to the manufacturer’s instructions. Briefly, 5 × 10⁶ cells were collected and lysed in extraction buffer. After sonication, samples were centrifuged at 8000 × *g* for 10 min at 4 °C. The resultant supernatants were collected for analysis. Acetyl-CoA levels were measured using a spectrophotometric method, which involves the conversion of acetyl-CoA to CoA and the subsequent formation of NADH, quantified by absorbance at 340 nm.

### ATP quantification

ATP levels were quantified using the ATP Assay Kit (Beyotime Biotechnology, #S0026) according to the manufacturer’s instructions. Briefly, cells were collected and lysed with ice-cold lysis buffer, and the supernatants were collected after centrifugation at 12 000 × g for 5 min at 4 °C. A standard curve was generated using serial dilutions of the provided ATP standard solution. Following a 5 min incubation of 100 μL ATP detection working solution at room temperature to consume intrinsic ATP background, 20 μL of sample or standard was added into each well and immediately mixed. Chemiluminescence was measured using a multifunctional microplate reader operating in luminescence mode. The ATP concentrations were calculated based on the standard curve and normalized to the total protein concentration determined by a BCA assay.

### ChIP

U2OS-DR-GFP cells were crosslinked with 1% formaldehyde, quenched with 100 mM glycine, then lysed sequentially in cell lysis buffer (85 mM KCl, 5 mM PIPES pH 8.0, 0.5% NP-40) and nuclei lysis buffer (10 mM EDTA, pH 8.0, 50 mM Tris-HCl pH 8.0, 1% SDS). Chromatin was sonicated to 200–500 bp fragments using a Vibra-Cell sonicator, clarified by centrifugation, and diluted in ChIP dilution buffer. Chromatin aliquots were immunoprecipitated overnight at 4 °C with rotation using specific antibodies. Immune complexes were captured with Protein G magnetic beads, washed sequentially with buffer Ⅰ (150 mM NaCl, 20 mM Tris-HCl pH 8.0, 2 mM EDTA pH 8.0, 1% Triton X-100, 0.1% SDS) and buffer Ⅱ (500 mM NaCl, 20 mM Tris-HCl pH 8.0, 2 mM EDTA pH 8.0, 1% Triton X-100, 0.1% SDS), then eluted in elution buffer (50 mM Tris-HCl pH 8.0, 10 mM EDTA pH 8.0, 1% SDS). Crosslinks were reversed by incubation with RNase and proteinase K. DNA was purified using a PCR purification kit and quantified by qPCR using DR-GFP Set9 primers: forward, 5′-ATCACATGGTCCTGCTGGAGTT-3′, reverse, 5′-TGGCTGATTATGATCTAGAGTCGCGG-3′.

### HR and NHEJ analysis

To assess HR and NHEJ efficiencies, U2OS cell lines stably integrated with DR-GFP or EJ5-GFP reporter systems were used, as previously established [47, 48]. Cells were transfected with an HA-tagged I-SceI expression vector using Lipofectamine 2000 for 48 h to induce site-specific DSBs. HR-mediated repair utilized a truncated 3′ GFP fragment as a template to restore functional GFP, while NHEJ repaired the I-SceI-induced frameshift through error-prone ligation. The frequencies of HR and NHEJ were quantified by flow cytometry through the detection of GFP-positive cells.

### Chromatin relaxation assay

The chromatin relaxation assay was performed as described previously [49]. Briefly, cells were harvested 4 h after 10 Gy IR, lysed in hypotonic buffer (10 mM Tris-HCl, pH 8.0, 10 mM MgCl_2_, 25% glycerol, 0.2% NP-40, 1 mM DTT) for 30 min, and centrifuged to pellet nuclei. Nuclei were resuspended in MNase digestion buffer (15 mM Tris-HCl, pH 7.4, 60 mM KCl, 15 mM NaCl, 0.25 M sucrose, 1 mM CaCl₂, 0.5 mM DTT) and treated with MNase (New England Biolabs, #M0247S) at 37 °C for 5 min. Reactions were terminated by adding stop solution (1% SDS, 20 mM EDTA). DNA was purified via phenol-chloroform extraction, and digested fragments were separated on a 1.2% agarose gel.

### Tumour xenografts

MDA-MB-231 cells transduced with lentiviruses expressing either control vector or Flag-tagged ACSS1 were orthotopically implanted into the mammary fat pads of 5-week-old female nude mice (5 × 10^6^ cells in 100 μL PBS per mouse). Each transfection group initially consisted of 8 mice. When the tumour volume reached approximately 80 mm³, mice within each group were randomly assigned into two subgroups: 4 mice were subjected to tumour irradiation with 5 Gy IR for three consecutive days, while the remaining 4 mice served as non-irradiated controls. No blinding was done to group allocation. Tumour dimensions were measured every two days with callipers, and volumes were calculated as (length × width²)/2. All protocols were approved by the Animal Ethics Committee of Tianjin Medical University.

### Immunohistochemistry

Tumour sections were deparaffinized, rehydrated, and underwent antigen retrieval using citrate buffer. Subsequently, sections were incubated overnight at 4 °C with the indicated primary antibodies, followed by incubation with secondary antibodies for 30 min at room temperature. The results were visualized using an inverted microscope, with DAB serving as the chromogenic substrate.

### TUNEL assay

Paraffin-embedded tumour sections were fixed using 4% paraformaldehyde and subsequently dewaxed. Apoptosis detection was then conducted through TUNEL staining employing the One Step TUNEL Apoptosis Assay Kit (Beyotime, #C1088) following the manufacturer’s protocol. Ultimately, the stained slides were examined and visualized under a fluorescence microscope.

### Quantitative proteomics by multiplexed tandem mass tag (TMT) LC-MS/MS

Protein extraction from cell samples involved sonication, centrifugation, and quantification via BCA assay. Samples were reduced, alkylated, and diluted before overnight trypsin digestion. For TMT labeling, peptides were desalted, vacuum-dried, reconstituted, and incubated with TMT reagents. HPLC fractionation was performed using a high pH reverse-phase column, resulting in 18 fractions. LC-MS/MS analysis involved a gradient elution on a homemade column, with peptides analyzed on a Q Exactive^TM^ Plus mass spectrometer. Database search was conducted using Maxquant, with strict FDR settings and specific modifications considered.

### Statistical analysis

Data are presented as mean ± SD from at least three independent biological replicates. Statistical analyses were performed using GraphPad Prism 8.3.0. For comparisons between two groups, a two-tailed unpaired Student’s *t*-test was applied. Comparisons among multiple groups were analyzed by ANOVA with Bonferroni’s correction.

For clinical data analysis, the expression profiles of ACSS1 and clinicopathological characteristics of breast cancer patients were retrieved from the TCGA-BRCA database and GEO datasets (GSE80999 and GSE210411). We used chi-square tests, Welch’s *t*-tests, and Yates’ correction to examine differences in baseline characteristics across groups. Additionally, survival prognosis analysis was performed using the Log-rank test, univariate, and multivariable Cox regression models. Differential gene expression and ACSS1 expression patterns across breast cancer subgroups were analyzed through Bayesian inference, linear regression, and nonparametric tests (Wilcoxon rank-sum and Kruskal-Wallis tests).

Statistical significance was defined as *P* < 0.05 (*P < 0.05, **P < 0.01, ***P < 0.001). All visualizations were generated using R packages including *survival*, *survminer*, *ggplot2*, *forestplot*, *limma*, *VennDiagram*, *stats*, *car*, and *clusterProfiler*.

## Supplementary information

Figure S1

Figure S2

Figure S3

Figure S4

Figure S5

Table S1

Supplemental Material

Supplemental Material

## Data Availability

All data are available from the corresponding authors upon reasonable request.
