# Supplementary figures and images for: ACSS1 co-opts acetyl-CoA metabolism to drive DNA repair and undermine radiotherapy efficacy in breast cancer
Source: Cell Death Dis. 2025 Dec 18;17(1):119. doi: 10.1038/s41419-025-08300-w (PMC12847742; doi:10.1038/s41419-025-08300-w)

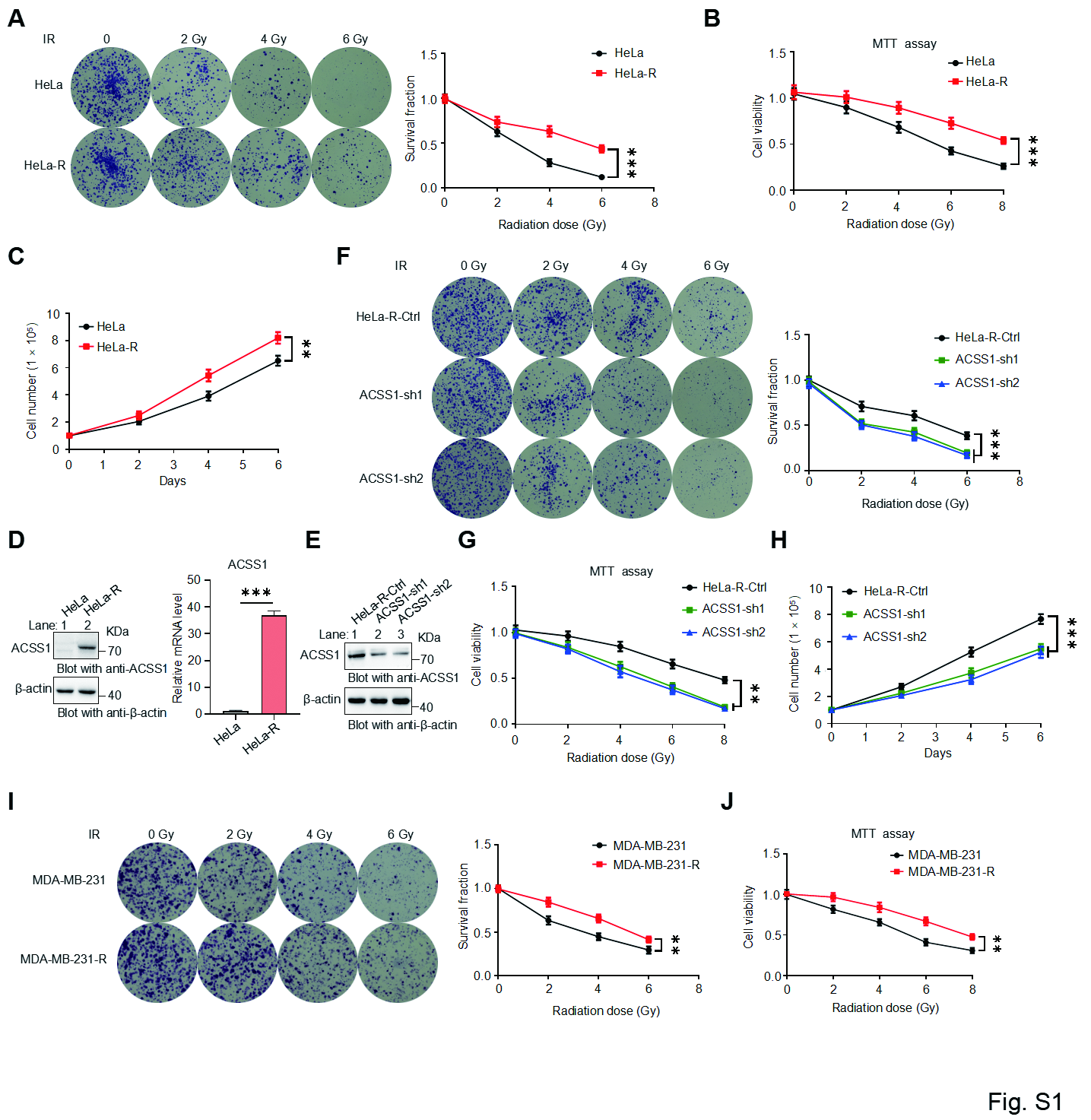

Supplement: Supplementary file 1 — Figure S1 [file 41419_2025_8300_MOESM1_ESM.tif]

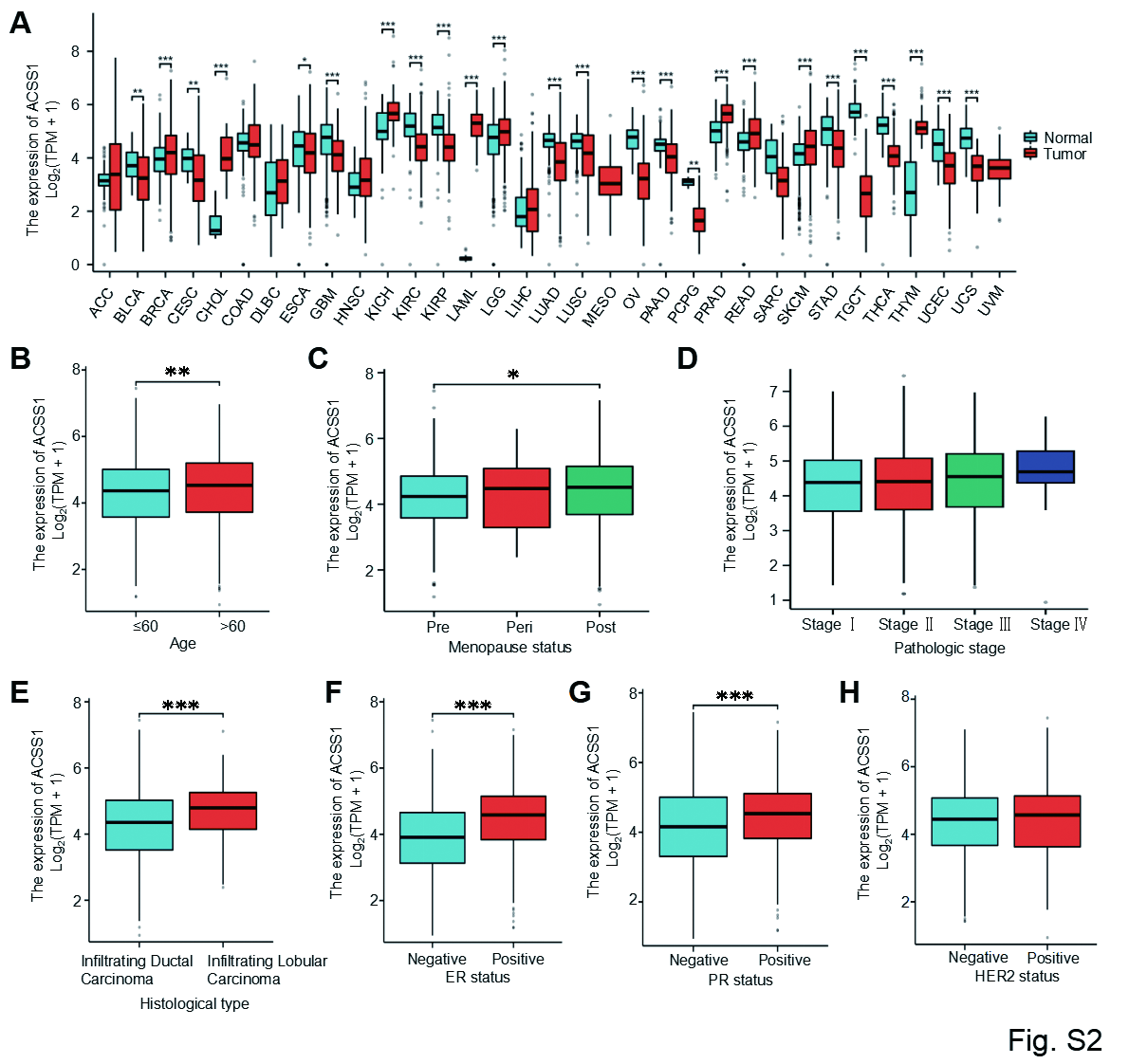

Supplement: Supplementary file 2 — Figure S2 [file 41419_2025_8300_MOESM2_ESM.tif]

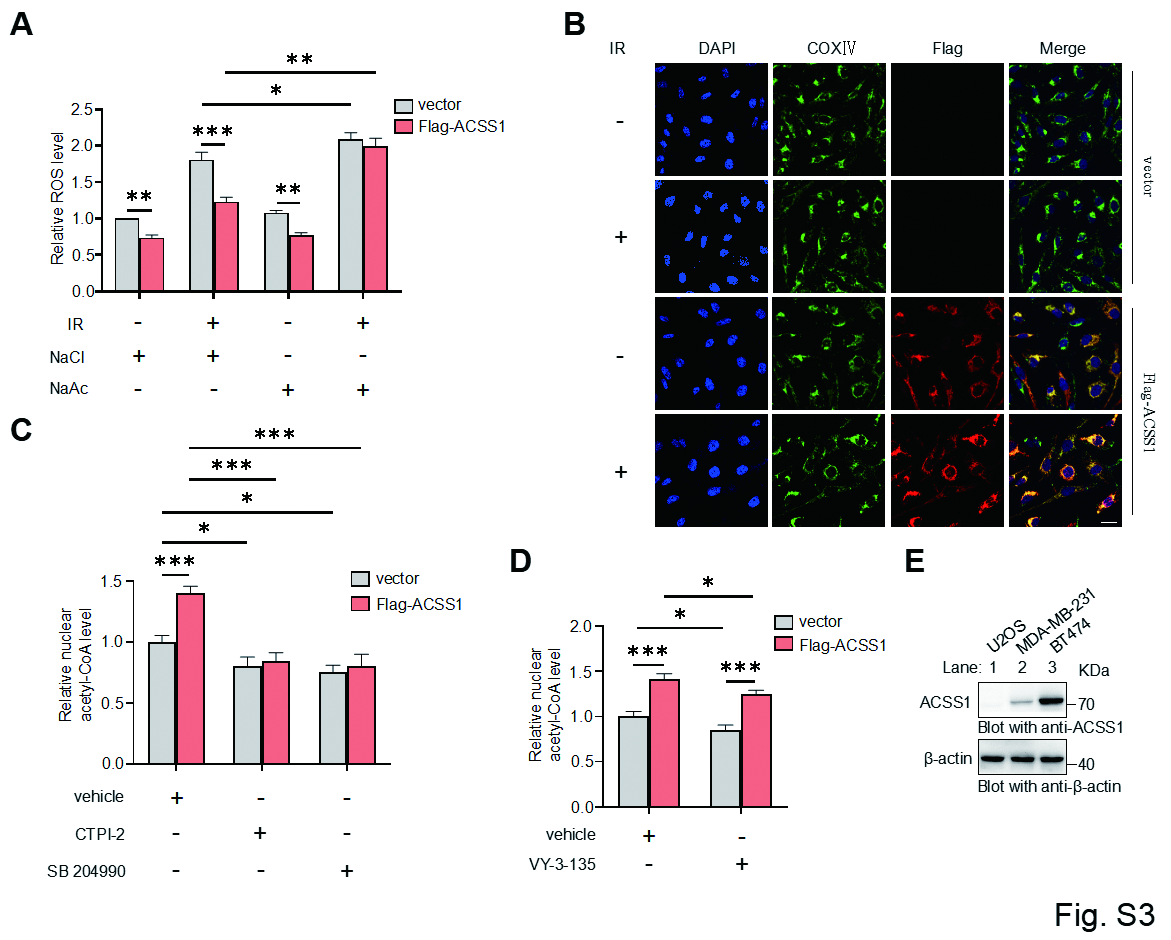

Supplement: Supplementary file 3 — Figure S3 [file 41419_2025_8300_MOESM3_ESM.tif]

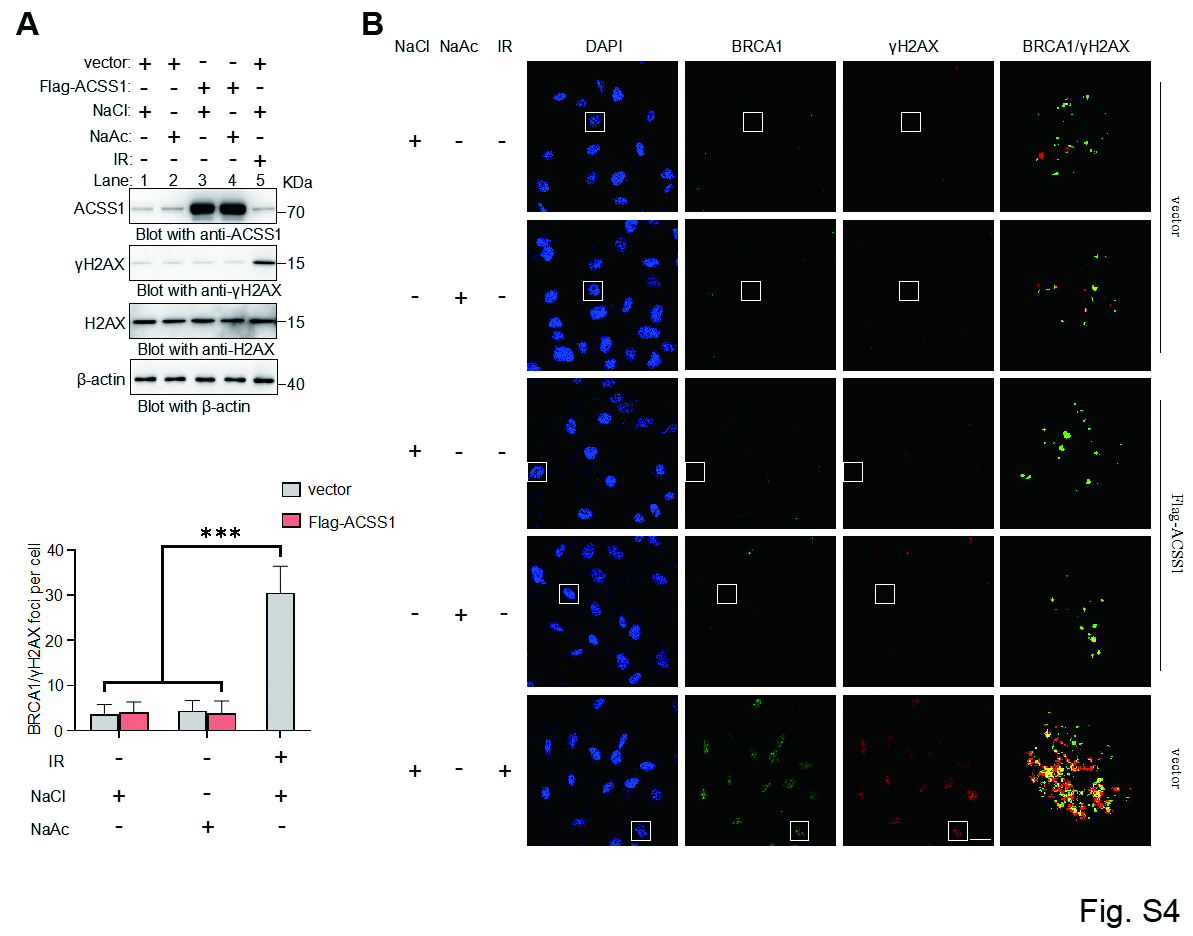

Supplement: Supplementary file 4 — Figure S4 [file 41419_2025_8300_MOESM4_ESM.tif]

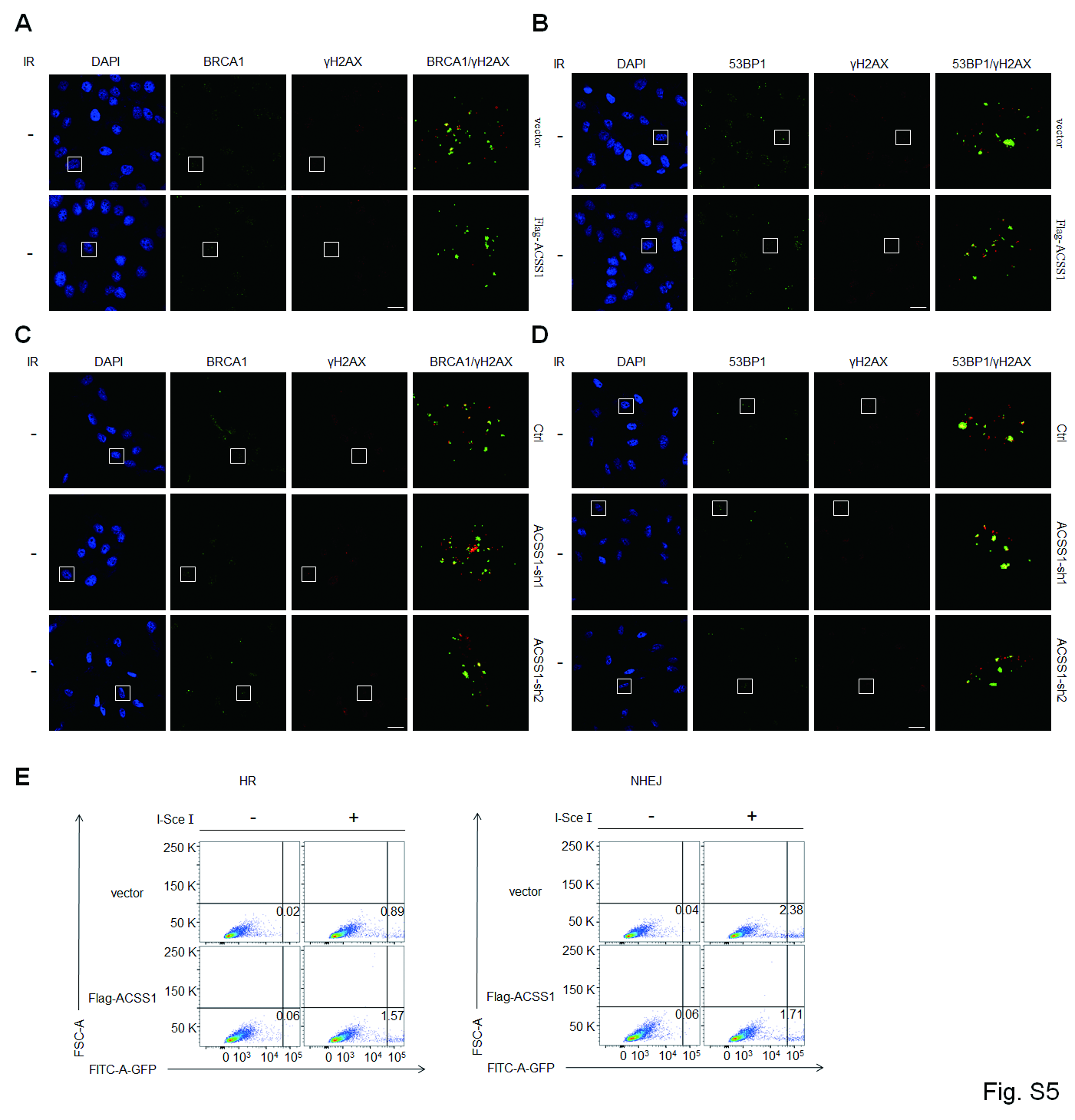

Supplement: Supplementary file 5 — Figure S5 [file 41419_2025_8300_MOESM5_ESM.tif]
