# Supplementary material for: ACSS1 co-opts acetyl-CoA metabolism to drive DNA repair and undermine radiotherapy efficacy in breast cancer
Source: Cell Death Dis. 2025 Dec 18;17(1):119. doi: 10.1038/s41419-025-08300-w (PMC12847742; doi:10.1038/s41419-025-08300-w)
Supplement: Supplementary file 7 — Supplemental Material [file 41419_2025_8300_MOESM7_ESM.docx]

**SUPPLEMENTARY FIGURE LEGENDS**

**Fig. S1 HeLa-R and MDA-MB-231-R** **cells exhibit increased resistance to radiation**

A, B Colony formation (A) and MTT assay (B) of HeLa-R cells treated with the indicated doses of radiation. C The cell growth viability was determined in parental and HeLa-R cells after 4 Gy IR treatment. D ACSS1 expression in parental and HeLa-R cells. Protein levels were analyzed by western blot and mRNA levels by RT-qPCR. E Expression level of ACSS1 in HeLa-R cells. F, G Colony formation (F) and MTT assay (G) of ACSS1-depleted HeLa-R cells exposed to the indicated doses of radiation. H Cell growth viability was determined in HeLa-R cells with a loss of ACSS1 function after 4 Gy IR treatment. I, J Colony formation (I) and MTT assay (J) of MDA-MB-231-R cells exposed to the indicated doses of radiation. Each bar represents the mean ± SD for triplicate experiments. Statistical analysis was performed using two-way ANOVA for A, B, C, F, G, H, I, and J; two-tailed unpaired Student’s *t*-test for D; ***P* < 0.01; ****P* < 0.001.

**Fig. S2 The correlation of ACSS1 protein expression with clinical characteristics in breast cancer patients**

A Pan-cancer analysis of ACSS1 differential expression across tumour types in the TCGA database. B-H Correlation analyses in the breast cancer cohort encompass: age (B), menopause status (C), pathologic stage (D), histological type (E), estrogen receptor (ER) status (F), progesterone receptor (PR) status (G), and human epidermal growth factor receptor 2 (HER2) status (H). Each bar represents the mean ± SD and statistical analysis was performed using Wilcoxon rank sum test for A, B, E, F, G, and H; Kruskal-Wallis test for C and D; **P* < 0.05; ***P* < 0.01; ****P* < 0.001.

**Fig. S3 ACSS1 regulates IR-induced metabolic dynamics and nuclear acetyl-CoA production via a citrate-ACLY axis**

A Supplementation with NaAc reduces the ACSS1-mediated decrease in ROS levels following IR. B Immunofluorescence assay confirms mitochondrial localization of Flag-ACSS1. C The ACSS1-dependent increase in nuclear acetyl-CoA is attenuated by inhibition of the citrate transporter (CTPI-2) or ACLY (SB 204990). D ACSS2 inhibition (VY-3-135) does not affect the ACSS1-overexpression-induced increase in nuclear acetyl-CoA. E Western blot showing negligible endogenous ACSS1 expression in U2OS cells. Each bar represents the mean ± SD for triplicate experiments. Statistical analysis was performed using two-way ANOVA;**P* < 0.05; ***P* < 0.01; ****P* < 0.001.

**Fig. S4 Acetate supplementation does not induce DNA damage response at baseline**

A Western blot analysis of γH2AX in cells treated with or without NaAc. B Immunofluorescence images and quantification of γH2AX and BRCA1 foci in cells treated with or without NaAc. Statistical analysis was performed using two-way ANOVA; ****P* < 0.001.

**Fig. S5 ACSS1 promotes HR-mediated repair**

A, C Immunofluorescence assay showing the number of BRCA1 foci in MDA-MB-231 (A) and BT474 cells (C) without IR treatment. Scale bar: 25 μm. B, D Immunofluorescence assay showing the number of 53BP1 foci in MDA-MB-231 (B) cells and BT474 cells (D) without IR treatment. Scale bar: 25 μm. E HR and NHEJ repair frequency was determined by flow cytometry.
