# Supplementary material for: ACSS1 co-opts acetyl-CoA metabolism to drive DNA repair and undermine radiotherapy efficacy in breast cancer
Source: Cell Death Dis. 2025 Dec 18;17(1):119. doi: 10.1038/s41419-025-08300-w (PMC12847742; doi:10.1038/s41419-025-08300-w)

Figure 2-E:

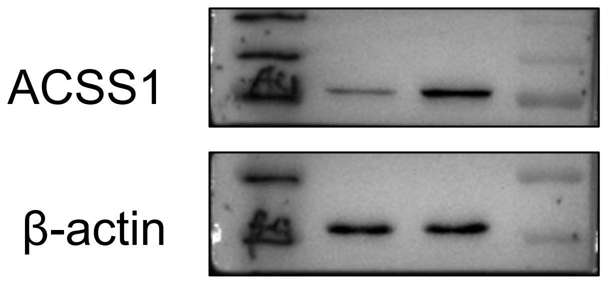


Figure 2-F:

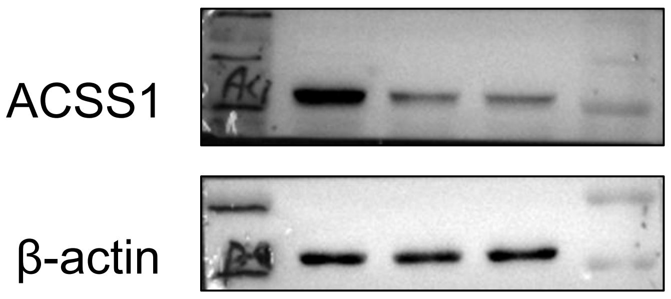


Figure 3-E:

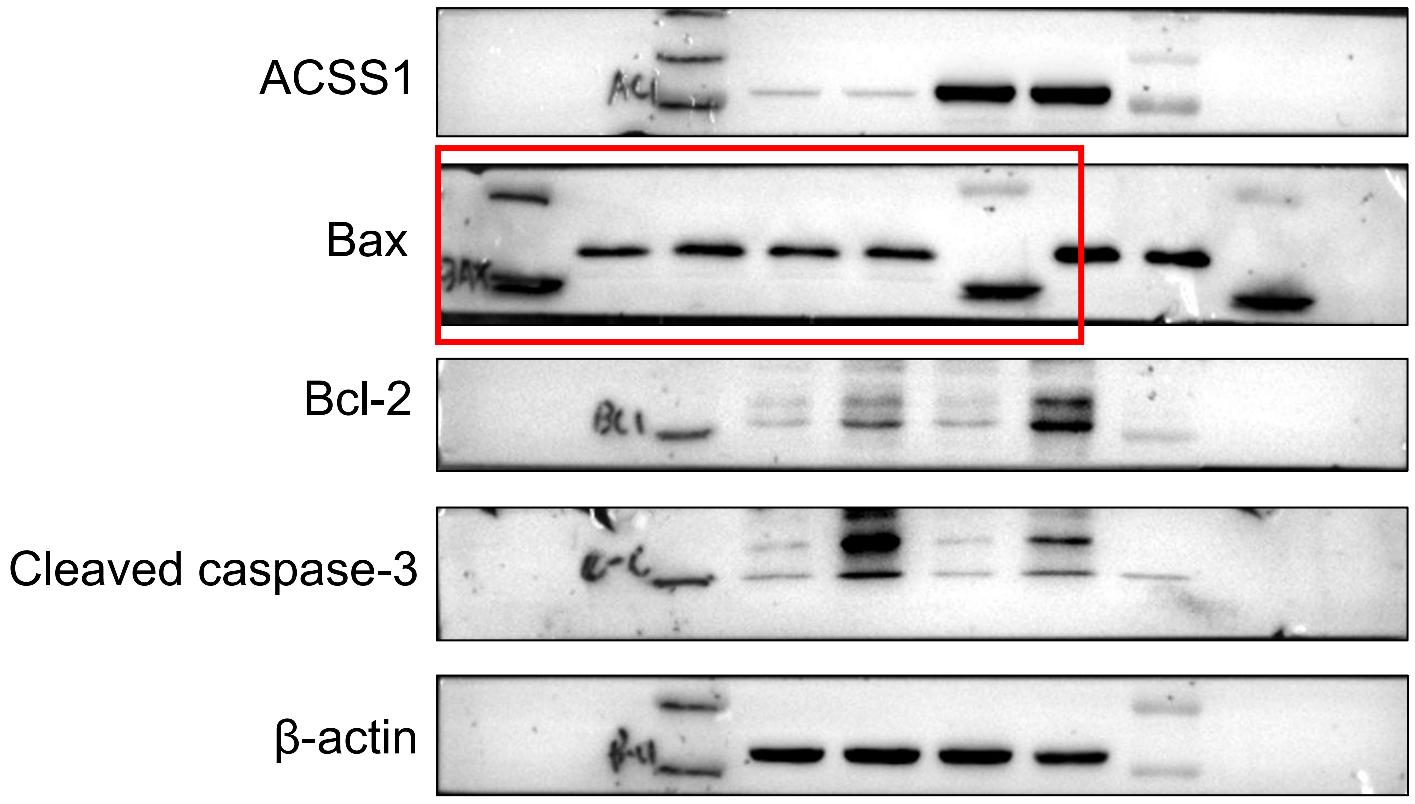


Figure 3-J:

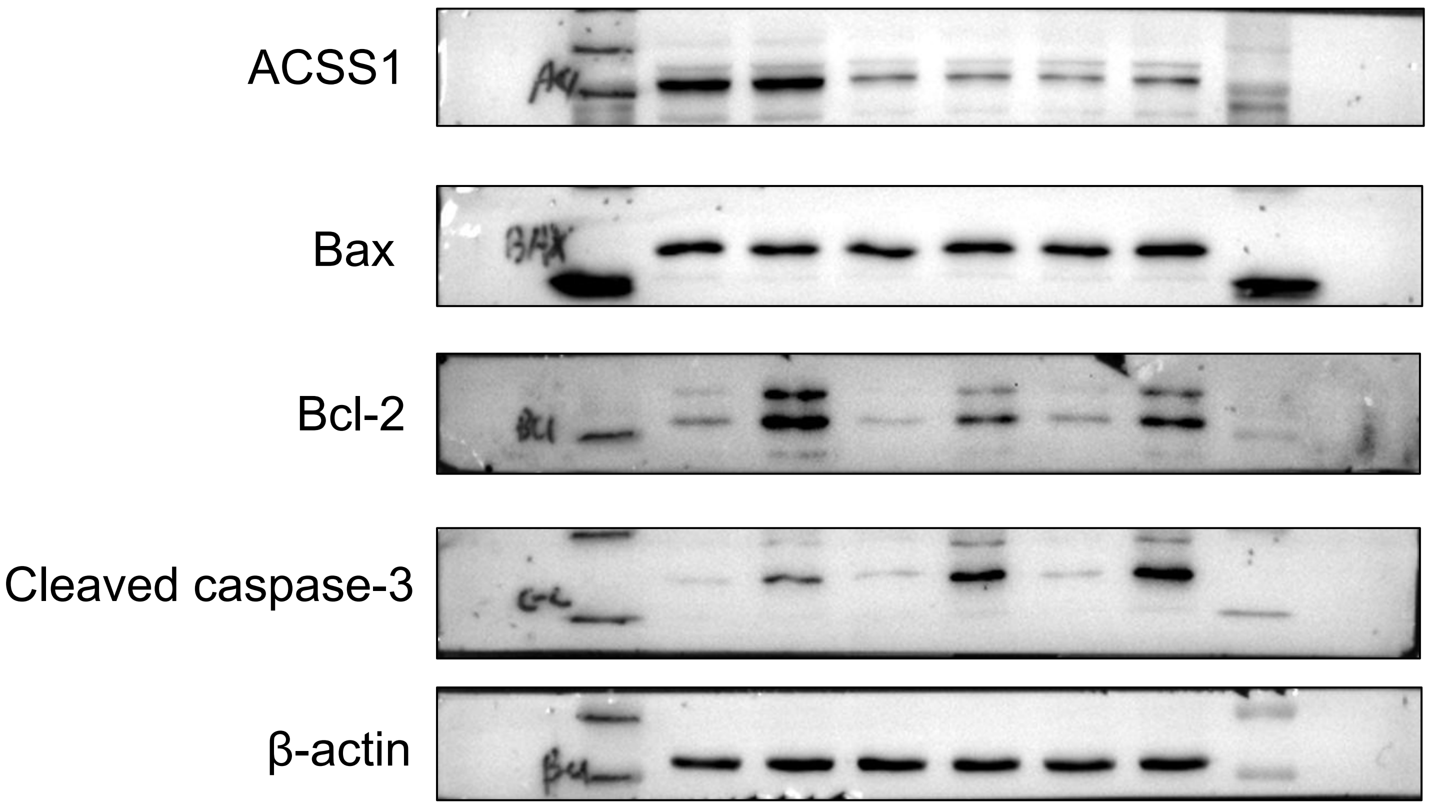

Figure 4-B:

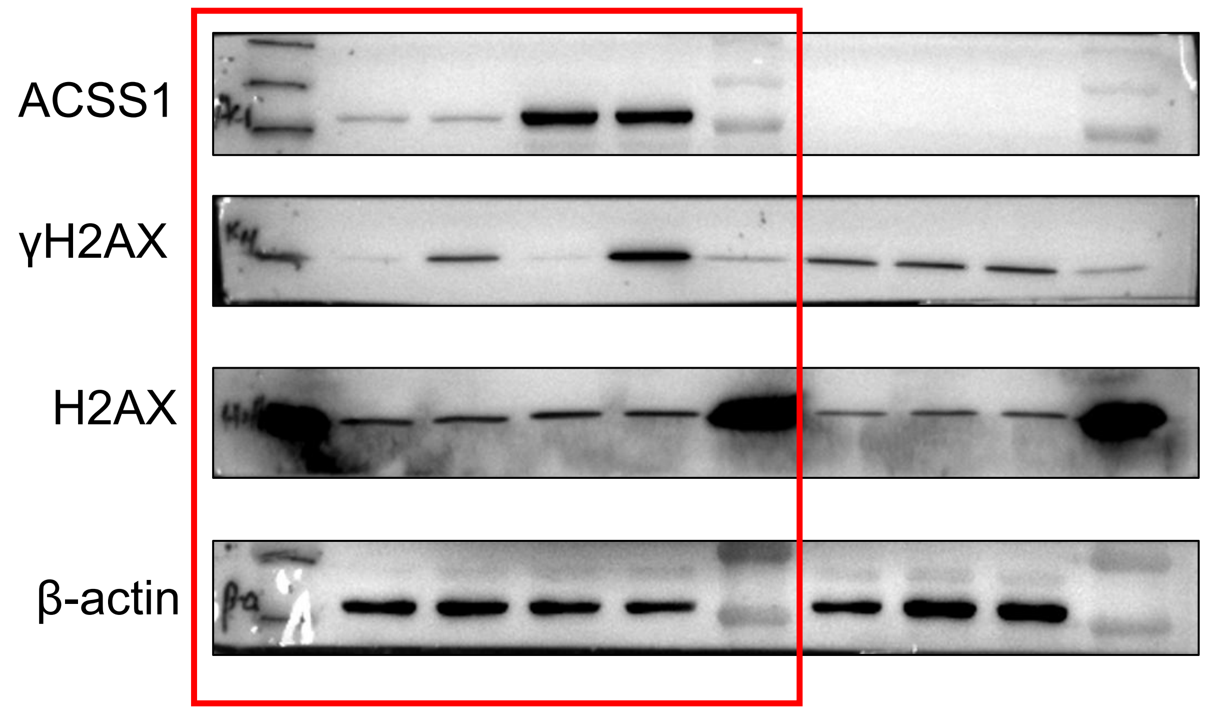


Figure 4-D:

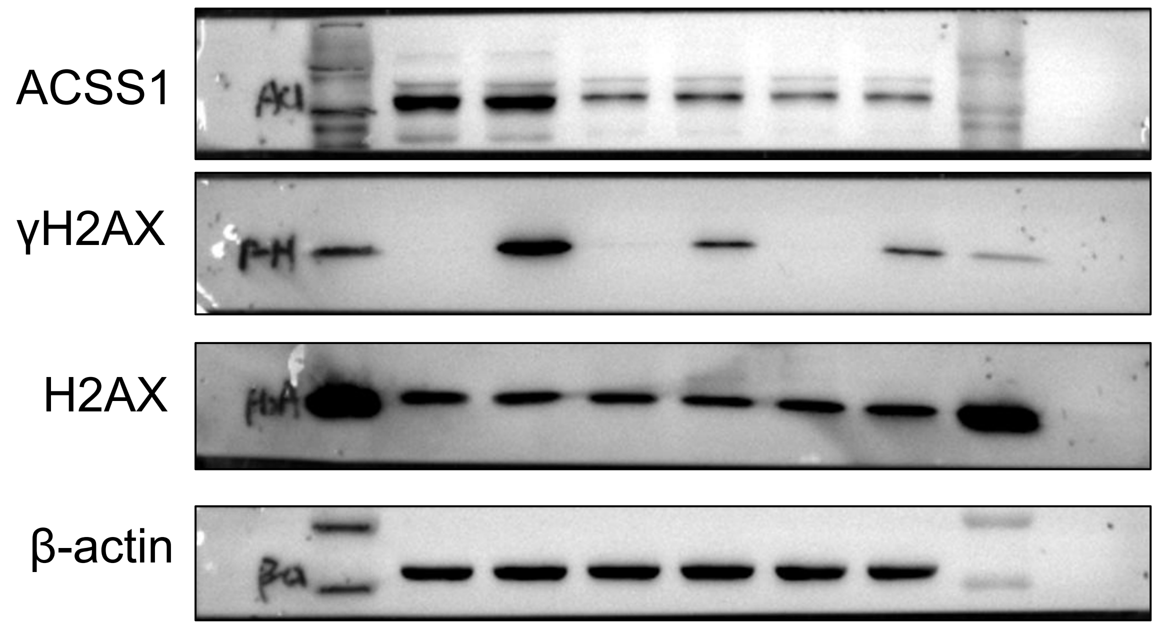


Figure 6-A:

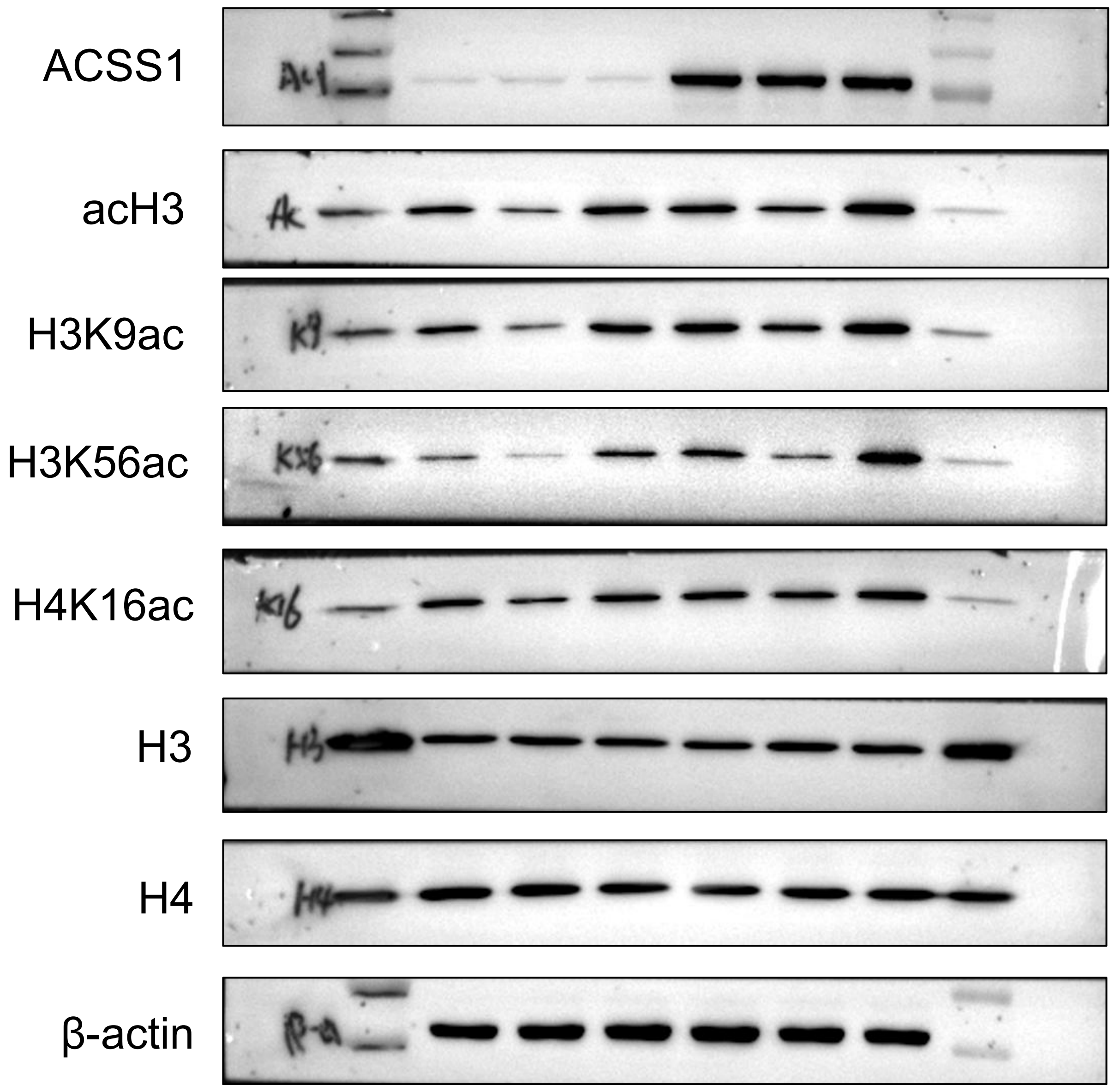

Figure 6-B:

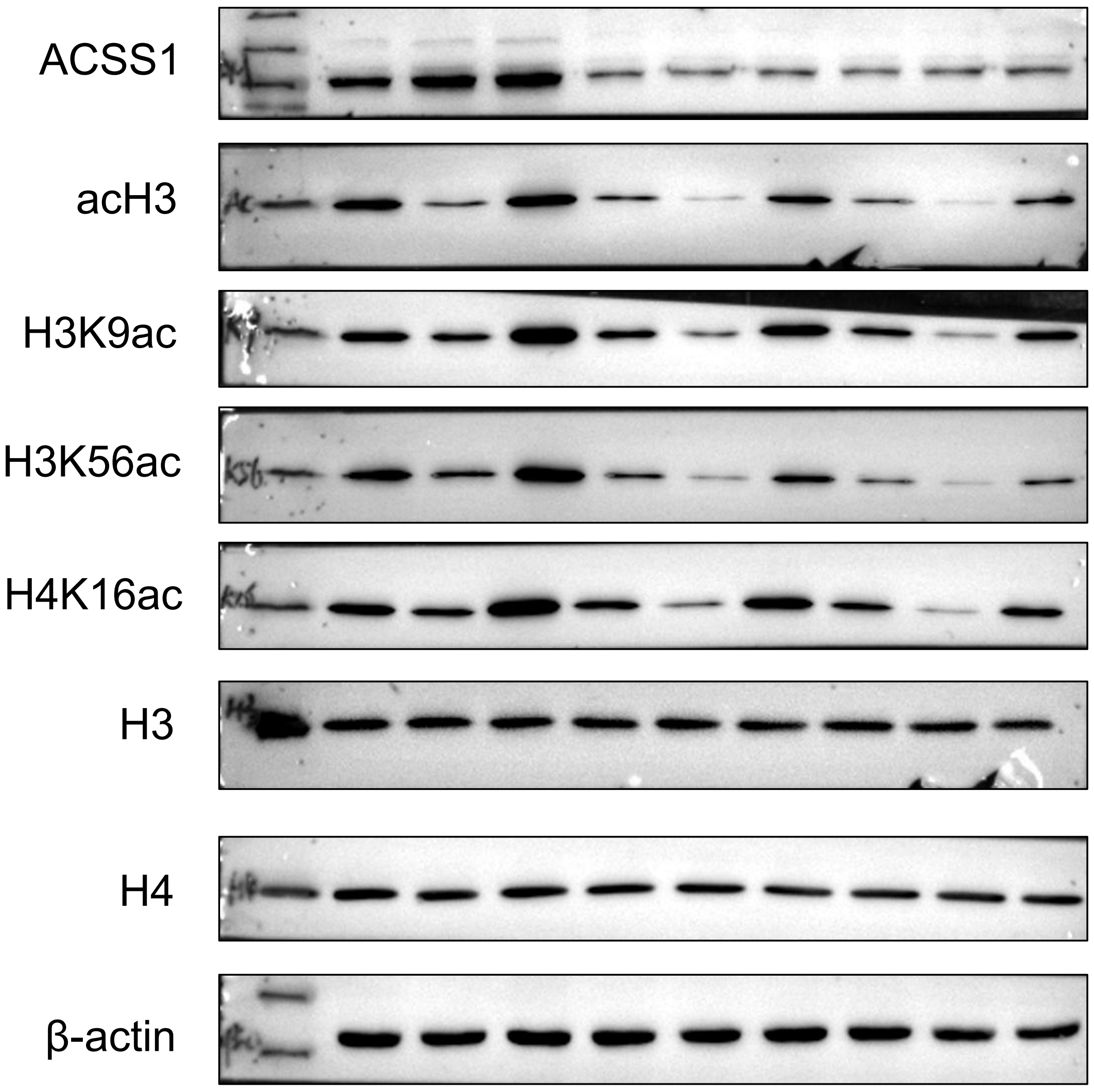


Figure 6-D:

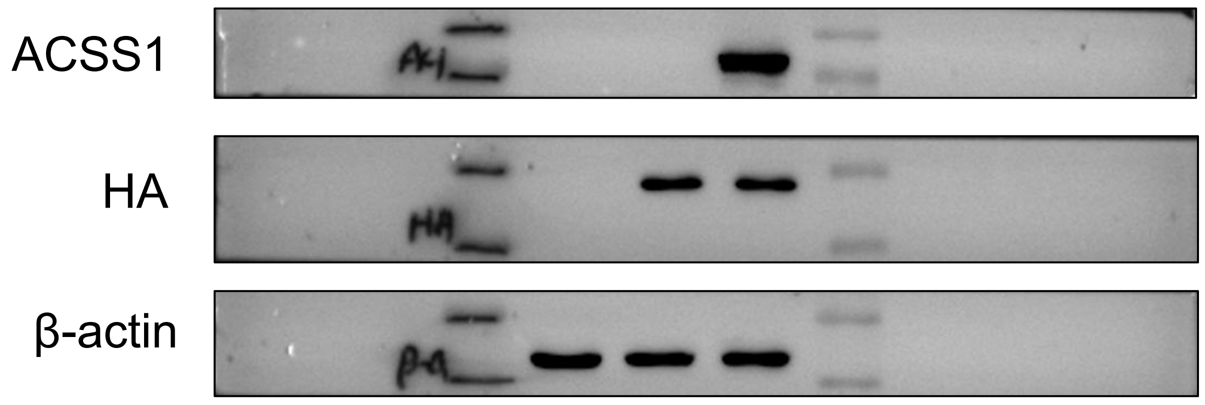


Figure 7-G:

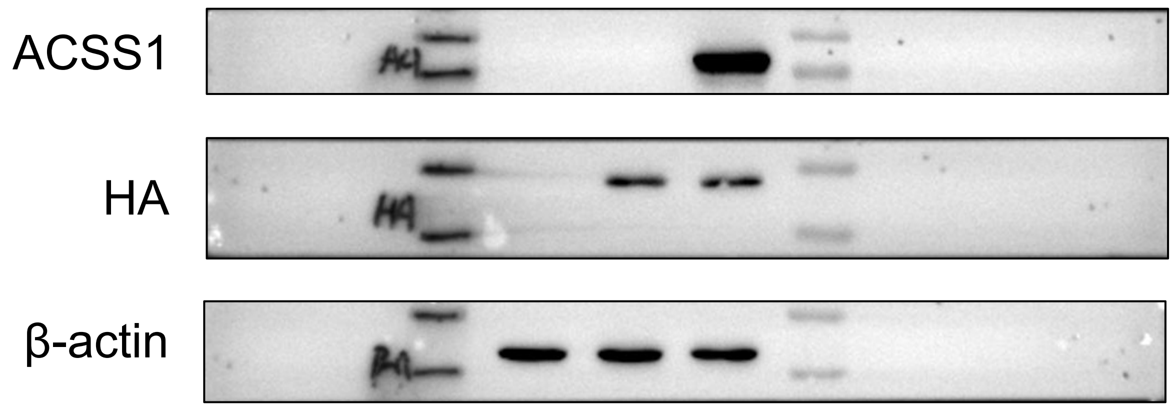


Figure 7-I:

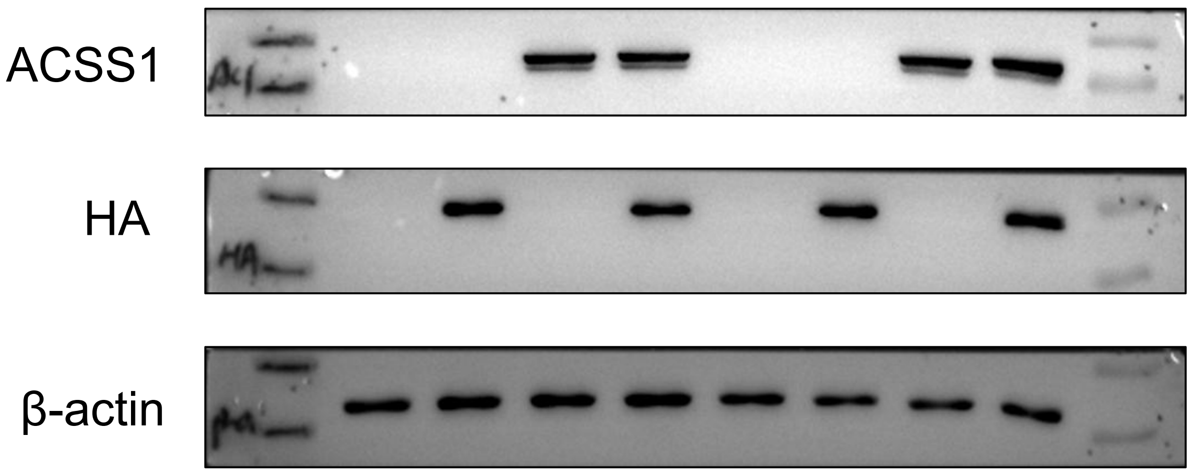


Figure 8-F:

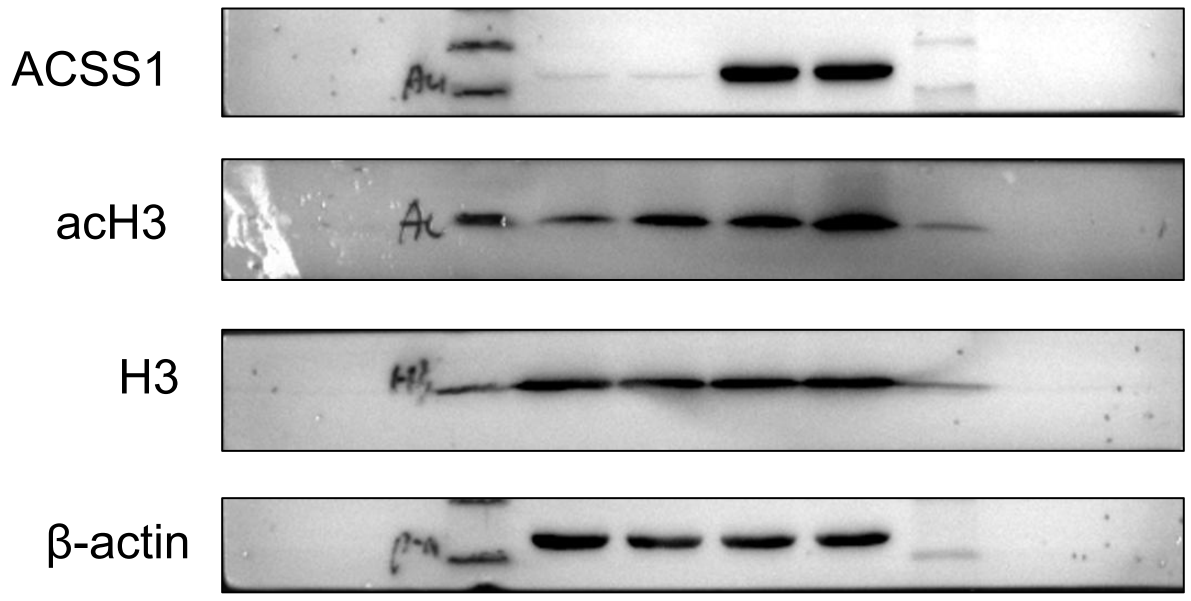

Figure S1-D:

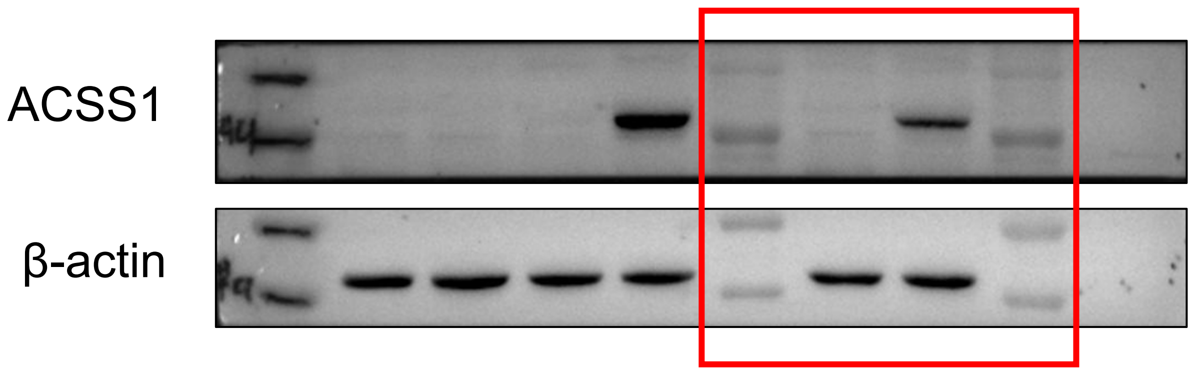


Figure S1-E:

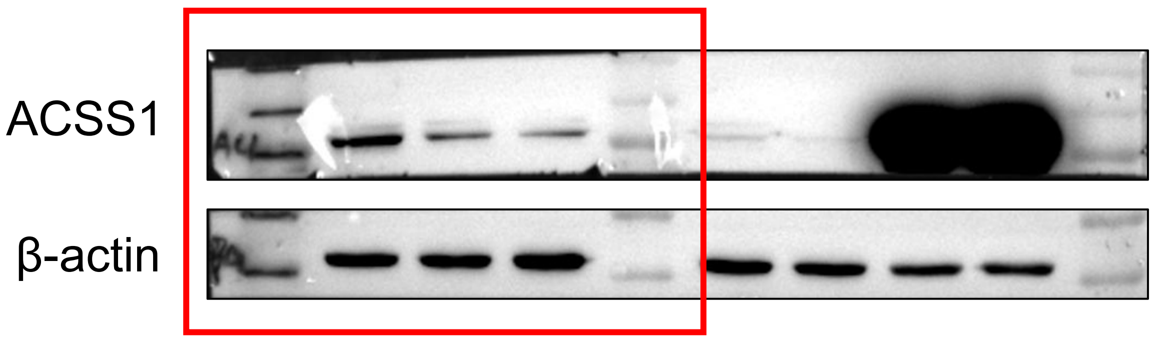


Figure S3-E:

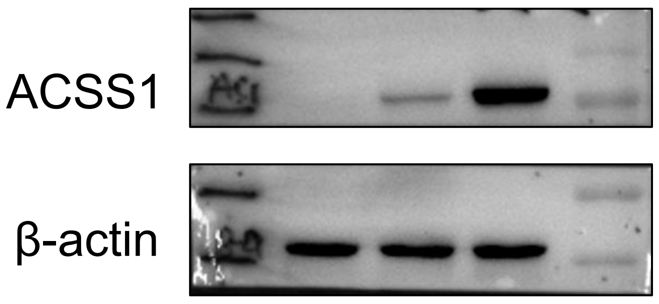


Figure S4-A:

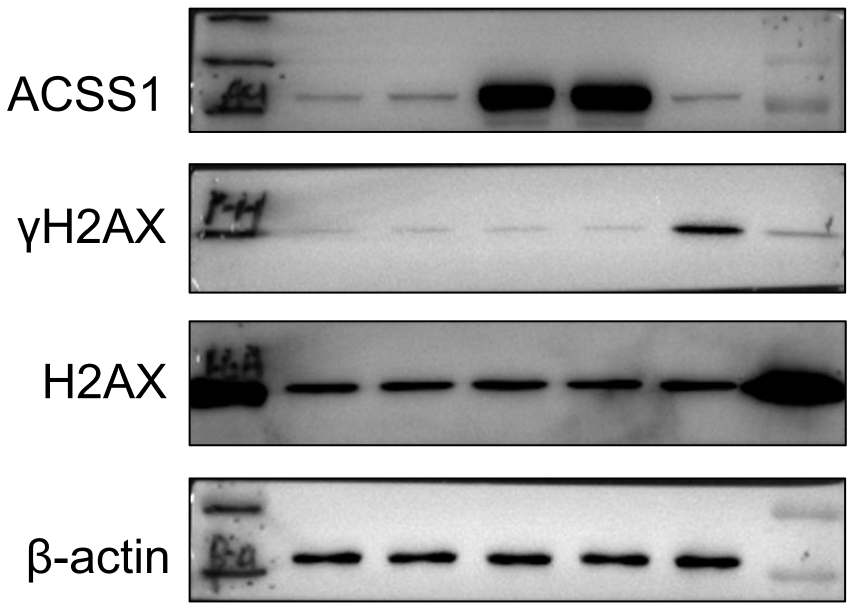

Supplement: Supplementary file 8 — Supplemental Material [file 41419_2025_8300_MOESM8_ESM.pdf]
